# Bioactive Novel Indole Alkaloids and Steroids from Deep Sea-Derived Fungus *Aspergillus fumigatus* SCSIO 41012

**DOI:** 10.3390/molecules23092379

**Published:** 2018-09-18

**Authors:** Salendra Limbadri, Xiaowei Luo, Xiuping Lin, Shengrong Liao, Junfeng Wang, Xuefeng Zhou, Bin Yang, Yonghong Liu

**Affiliations:** 1 Key Laboratory of Tropical Marine Bio-resources and Ecology/Guangdong Key Laboratory of Marine Materia Medica/Research Center for Marine Microbes, South China Sea Institute of Oceanology, Chinese Academy of Sciences, Guangzhou 510301, China; raj.badri202@gmail.com (S.L.); luoxiaowei14@mails.ucas.ac.cn (X.L.); sophielxp@163.com (X.L.); ljrss@126.com (S.L.); junfeng1982a@163.com (J.W.); xfzhou@scsio.ac.cn (X.Z.); 2 University of Chinese Academy of Sciences, Beijing 100049, China

**Keywords:** deep sea-derived fungus, *Aspergillus fumigatus* SCSIO 41012, indole alkaloids, steroids, antibacterial activity, antifungal activity

## Abstract

Two new alkaloids, fumigatosides E (**1**) and F (**2**), and a new natural product, 3, 7-diketo-cephalosporin P_1_ (**6**) along with five known compounds (**3**–**5**, **7**, **8**) were isolated from deep-sea derived fungal *Aspergillus fumigatus* SCSIO 41012. Their structures were determined by extensive spectroscopic data analysis, including 1D, 2D nuclear magnetic resonance (NMR) and mass spectrometry (MS), and comparison between the calculated and experimental electronic circular dichroism (ECD) spectra. In addition, all compounds were tested for antibacterial and antifungal inhibitory activities. Compound **1** showed significant antifungal activity against *Fusarium oxysporum* f. sp. *momordicae* with MIC at 1.56 µg/mL. Compound **4** exhibited significant higher activity against *S. aureus* (16,339 and 29,213) with MIC values of 1.56 and 0.78 µg/mL, respectively, and compound **2** exhibited significant activity against *A. baumanii* ATCC 19606 with a MIC value of 6.25 µg/mL.

## 1. Introduction

Marine fungi are known to be rich sources of biologically active compounds for medicinal and agricultural applications [1,2,3,4]. Indole alkaloids have attracted a great deal of attention due to their diversified structures and potent biological activities, such as cytotoxicity, anti-feedant, and anti-microbial activities [5,6]. In recent years, marine-derived fungi have been demonstrated to be a rich and promising source of indole alkaloids [7,8]. It is notable that indole alkaloids biogenetically synthesized by tryptophan were isolated from a diverse group of fungi, including the genera *Aspergillus, Chaetomium*, and *Penicillium* [8,9,10,11].

**Figure 1:**
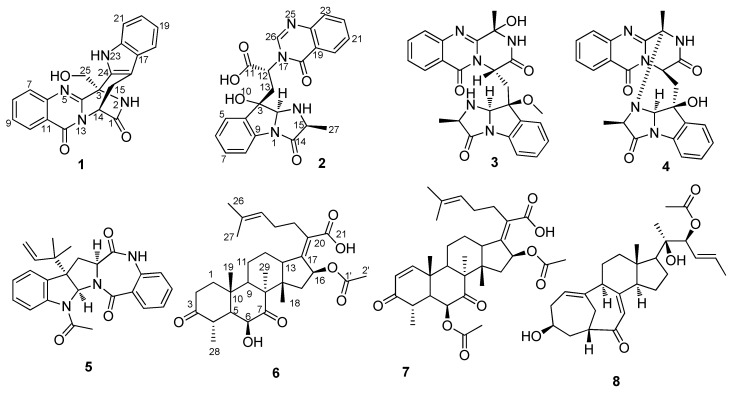
Structures of metabolites **1**–**8**.

The genus *Aspergillus* is one of the largest and most intensively investigated fungal genera. Previous investigations by our group into *Aspergillus* species, derived from the jellyfish *Nemopilema nomurai*, resulted in the isolation of three new pyrazinoquinazoline indole glucosides [12]. As a part of our continuing program to explore the antiviral potential of marine fungi, secondary metabolites of the SCSIO 41012 strain were examined. Two new compounds: Fumigatosides E (**1**) and F (**2**), together with four known indole alkaloids: Fumiquinazoline C (**3**), fumiquinazoline G (**4**) and epi-aszonalenin A (**5**) were isolated from the ethyl acetate crude extracts of rice medium. New sterols have rarely been discovered from microorganisms, such as fungi or actinomycetes [13]. A new natural sterol, 3,7-diketo-cephalosporin P_1_ (**6**), together with two known sterols, helvolic acid (**7**) and 22-*O*-acetylisocyclocitrinol A (**8**) (Figure 1) were also isolated. Their structures were established on the basis of extensive spectroscopic techniques. We present herein the fermentation, isolation, structural elucidation, and bioactive assay of compounds **1**–**8**.

## 2. Results

**Figure 2:**
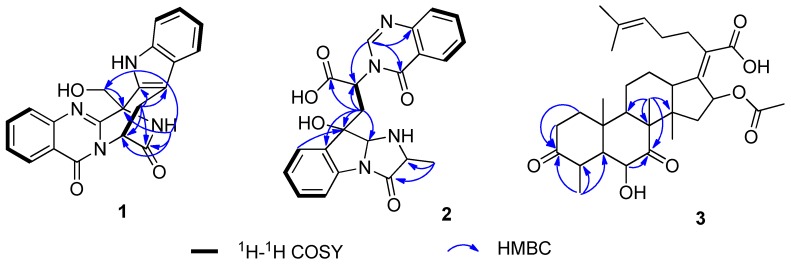
Key HMBC and ^1^H-^1^H homonuclear correlated spectroscopy (COSY) correlations of compounds **1** and **2.**

**Figure 3:**
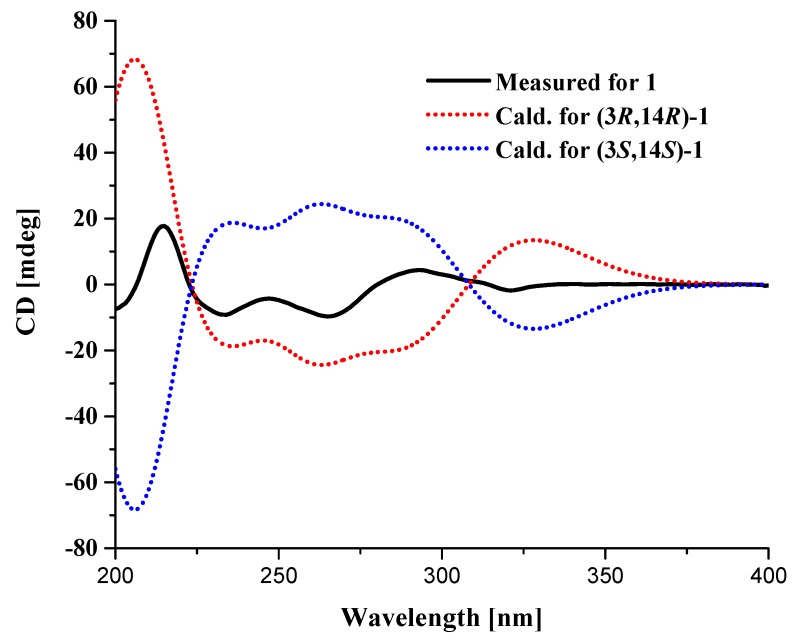
Comparison of calculated and experimental ECD spectra of **1**.

Compound **1** was isolated as a white amorphous solid. Based on the high resolution electrospray ionization mass spectroscopy (HRESIMS) ion peak at *m*/*z* 371.1153 [M − H]^−^ (calcd for 371.1150), the molecular formula was established as C_21_H_16_N_4_O_3_ indicating eleven degrees of unsaturation (Figure S9). The ^1^H NMR spectrum showed two sets of adjacent aromatic protons at *δ*_H_ 8.15 (dd, 8.4, 1.4), 7.83 (td, 8.4, 1.4), 7.55 (td, 8.4, 1.4), and 7.67 (d, 8.4) and *δ*_H_ 7.40 (d, 7.0), 7.12 (td, 7.0, 0.7), 6.99 (t, 7.0), and 7.41 (d, 7.0), suggesting the existence of two ortho-disubstituted aromatic rings. In addition, the ^1^H NMR spectrum showed signals corresponding to two methenes (*δ*_H_ 4.65 (s); 3.42 (dd, 17.5, 2.8); 3.24 (dd, 17.5, 4.9)), one methine (*δ*_H_ 5.69 (t, 17.5, 2.8)). The ^13^C NMR, distortionless enhancement by polarization transfer (DEPTs) and heteronculear single quantum coherence (HSQC) spectra (Table 1, Supplementary Materials) revealed the presence of two amide carbonyls (*δ*_C_ 169.4, 159.8), seven quaternary sp^2^ (*δ*_C_ 153.3, 147.0, 135.7, 132.5, 127.0, 120.7, 106.5), eight methine sp^2^ (*δ*_C_ 135.3, 127.8, 127.7, 126.8, 122.7, 119.8, 118.5, 112.4), one quaternary sp^3^ (*δ*_C_ 58.4), one methine sp^3^ (*δ*_C_ 54.3), and two methylene sp^3^ (*δ*_C_ 60.4, 26.1). These spectral characteristics were indicative of a quinazoline containing indole alkaloid skeleton. By detailed 1D and 2D NMR analysis, the compound was found to be closely related to fumiquinazoline J [14]. The only difference between these two compounds is the presence of the oxygenated methine at C-3 in **1** instead of the methyl group of the latter. This was confirmed by the heteronuclear multiple bond correlation (HMBC) correlations of H-25 to C-3, NH-2 to C-1, C-3 C-14, and C-25, and H-15 to C-1, C-14, C-16, and C-24 (Figure 2). Meanwhile, the absolute configuration of compound **1** was determined as 3R, and 14R by time-dependent density functional theory electronic circular dichroism (TDDFT-ECD) calculations, which were carried out with the lowest-energy conformer of the truncated structure and provided simulated ECD spectra closely similar to the measured one (Figure 3, Supplementary Materials). Therefore, the structure of compound **1** was identified and given the trivial name fumigatoside E.

Compound **2** was isolated as a white amorphous solid. Based on the HRESIMS ion peak at *m*/*z* 419.1364 [M − H]^−^ (calcd for 419.1361), the molecular formula was established as C_22_H_20_N_4_O_5_ indicating fifteen degrees of unsaturations (Figure S18). The 1D and 2D NMR spectra (Table 1, Supplementary Materials) revealed the presence of two 1,2-disubstituted benzene rings of the *gem*-methyl imidazoindolone ring system and quinazolin-4(3*H*)-one moiety, similar to those of tryptoquivaline F [15]. Interestingly, C-3 (*δ*_C_ 90.9), C-11 (*δ*_C_ 172.2), and C-13 (*δ*_C_ 32.7) resonated at higher chemical shift values than their counterparts in tryptoquivaline F (*δ*_C_ 86.2, 170.8 and 30.7) while C-2 (*δ*_C_ 82.8), C-14 (*δ*_C_ 172.2) and C-15 (*δ*_C_ 58.0) exhibited lower chemical shift value than the corresponding carbon (*δ*_C_ 84.5, 176.8, and 59.5) of tryptoquivaline F. This implied that the five-membered spirolactone ring is opening, as is evident from the molecular formula of compound **2** (C_21_H_16_N_4_O_3_). Based on the chemical shift value of H-12 (*δ*_H_ 5.50, t, *J* = 14.0), the configuration of C-12 was the same as that of tryptoquivaline F [15,16]. The HMBC correlations shown in Figure 2 were used to construct the planar structure of **2**. In the nuclear overhauser effect spectroscopy (NOESY) spectrum of **2**, H-2 showed NOE with H-15, but not with H_3_-27, exhibited different correlations to those observed for tryptoquivalines L and H. The absolute configuration of compound **2** remains to be established.

Compound **6** was isolated as a white amorphous solid. Based on the HRESIMS ion peak at *m*/*z* 551.2968 [M + Na]^+^ (calcd for 551.2979), the molecular formula was established as C_31_H_44_O_7_ indicating ten degrees of unsaturation (Figure S28). Analysis of the ^13^C NMR (Table 2, Supplementary Materials) for **6** revealed five sp^3^ methyls and two olefinic methyls, seven methylenes, one trisubstituted and one tetrasubstituted double bonds, six methines including four sp^3^ methines and two sp^3^ methines linked to an oxygen atom, three sp^3^ quaternary carbons, and four carbonyl carbon. These data showed great similarities to those of 6-deacetyl-3-ketocephalosporin P_1_ [17,18], except for a ketone carbon instead of an oxygenated methine in C-7. This assumption was supported by the correlations of CH_3_-29 to C-7, C-8, C-9, and C-14, H-6 to C-7, and H-1 to C-3, H-2 to C-3 and CH_3_-28 to C-3, C-4, and C-5 in the HMBC spectrum (Figure 2). Therefore, **6** was characterized as 3, 7-diketo-cephalosporin P_1_. The similar CD profiles and same sign of optical rotations of two compounds suggested that the absolute configuration of **6** was the same as those of **7**.

By comparing the ^1^H, ^13^C NMR and MS data with the literature values, the known compounds were identified as, fumiquinazoline C (**3**) [19], fumiquinazoline G (**4**) [19], epi-aszonalenin A (**5**) [9,20], helvolic acid (**7**) [21,22], 22-*O*-acetylisocyclocitrinol A (**8**) [23,24]. The stereo-genic carbons of **3** and **8** were determined by an X-ray crystallographic analysis.

All the compounds (**1**–**8**) were tested for antibacterial and antifungal activities against five bacterial (*A. baumanii* ATCC 19606, *A. baumanii* ATCC 15122, *S. aureus* ATCC 16339, *S. aureus* ATCC 29213, and *K. pneumonia* ATCC 14578) and two fungal strains (*Fusarium oxysporum* f. sp. *cucumerinu* and *Fusarium oxysporum* f. sp. *momordicae*). All tested compounds exhibited moderate to high antimicrobial activity with MIC values ranging from 1.5 to 25 µg/mL as seen in Table 3. Streptomycin and nystatin were used as a positive control.

## 3. Materials and Methods

### 3.1. General Experimental Procedures

^1^H-, ^13^C-NMR, DEPT and 2D-NMR spectra were recorded on a Bruker AC 500 NMR spectrometer with tetramethylsilane (TMS) as an internal standard. HR-ESI-MS data were measured on a Bruker microTOF-QII mass spectrometer. CD spectra were measured with a Chirascan circular dichroism spectrometer (Applied Photophysics, Surrey, UK). Optical rotation values were measured with a PerkineElmer 341 polarimeter. Column chromatography was performed on silica gel (200–300 mesh; Qingdao Marine Chemical Factory, Qingdao, China), YMC gel (ODS-A, 12 nm, S-50 µm) and Sephadex LH-20 (Amersham Biosciences, Uppsala, Sweden), respectively. The silica gel GF_254_ used for TLC were supplied by the Qingdao Marine Chemical Factory, Qingdao, China. All solvents used were of analytical grade (Tianjin Fuyu Chemical and Industry Factory, Tianjin, China). HPLC was carried on Hitachi L-2400 with YMC ODS column. Spots were detected on TLC under UV light or by heating after spraying with 5% H_2_SO_4_ in EtOH (*v*/*v*).

### 3.2. Fungal Material

The fungal strain SCSIO 41012 was isolated from deep-sea sediments, which were collected from the Indian Ocean (Lat: 7°9.43716667′ N, Long: 89°4.4266667′ E) at a depth of 3614 m, in 2013. The isolated fungal strain was stored on Muller Hinton broth (MB) agar (malt extract 15 g, agar 15 g, sea-salt 10 g, water 1 L, and pH 7.4–7.8) slants at 4 °C, and was deposited in the CAS Key Laboratory of Tropical Marine Bio-resources and Ecology, South China Sea Institute of Oceanology, Chinese Academy of Sciences, Guangzhou, China. The fungal strain was identified by analysis of ITS region of the rDNA was described in Supplementary Materials. The resulting sequence data were similar to the sequence of *Aspergillus* sp. MBL1612 (accession no. KM924435), and was deposited in GenBank (accession no. KJ567462). Hence, the fungal strain was identified and named as *Aspergillus fumigatus* SCSIO 41012.

### 3.3. Fermentation and Extraction

The fungal strain *A. fumigatus* SCSIO 41012 was cultured on MB agar plates at 25 °C for one week, and cultured in 100 mL flasks (×48) each containing 10 mL of seed medium (malt extract: 15 g, sea salt: 2 g, distilled water: 1 mL and pH: 7.4–7.8) at 27 °C on rotary shakers (180 rpm) for 3 days. After fermentation fungal culture was carried out using solid rice medium (rice: 200 g/flask, sea salt: 2 g/flask and distilled water: 200 mL/flask), at 25 °C for 35 days. Later, fermented culture was harvested and extracted with acetone then filtered with cheese cloth to separate into filter solution and mycelia. The acetone filter solution was evaporated using a rotary vapor under reduced pressure to afford crude extract, and then extracted three times with ethyl acetate (EtOAc), while the mycelia was also extracted three times with EtOAc. The two ethyl acetate solutions were combined and evaporated under reduced pressure to afford a crude extract. The extract was suspended in a mixture of methanol and petroleum ether (1:1 *v*/*v*) to separate the oil from the crude. Finally, the methanol solution was concentrated under reduced pressure to yield 220 g of black crude extract.

### 3.4. Isolation and Purification

The black crude extract (220 g) was subjected to medium pressure liquid chromatography (MPLC) using silica gel and eluted with CH_2_Cl_2_/MeOH in gradient eluent (*v*/*v*: 100:0, 98:2, 97:3, 95:5, 90:10, 80:20, 50:50) to six fractions were obtained (fractions 1–6) based on thin layer chromatography (TLC). Fraction-2 (2 g) was further subjected to Sephadex LH-20 using methanol and then ultra-purified by semi-preparative reversed-phase HPLC (3 mL/min, UV detector λ_max_ 210 and 230 nm, CH_3_CN/H_2_O 50:50) to yield **1** (4 mg), and **2** (5 mg). Fraction 3 (4 g) was further subjected to Sephadex LH-20 (CH_2_Cl_2_/MeOH: 1:1) to obtained three sub-fractions. Fraction 3.1 (2 g) was purified by a silica gel column chromatography (CC) with petroleumether/ethylacetate gradient system (P.E/EtOAc: from 1.0 to 0.1) and then purified by semi-preparative reversed-phase HPLC (2 mL/min, UV detector λ_max_ 210 and 230 nm, MeOH/H_2_O 28:72) to yield **5** (14 mg), Fraction 3.2 (1 g) was subjected to silica gel column chromatography (CC) with CH_2_Cl_2_/MeOH gradient system (from 1.0 to 0.1) then purified by semi-preparative reversed-phase HPLC (2 mL/min, UV detector λ_max_ 210 and 230 nm, CH_3_CN/H_2_O 35:65) to yielded **3** (7 mg), **6** (18 mg). Fraction 5 was further subjected to Sephadex LH-20 (CH_2_Cl_2/_MeOH: 1:1) to two sub-fractions. Fraction 5.1 was purified by semi-preparative reversed-phase HPLC (3 mL/min, UV detector λ_max_ 210 and 230 nm, MeOH/H_2_O 30:70) to yield **4** (7 mg), **7** (16 mg) and **8** (8 mg).

Fumigatoside E (**1**)*.* White amorphous solid; UV (MeOH) λ_max_ (log ε): 202 (0.7), 220 (0.62), 230 (0.45) nm; IR (KBr) *V*_max_: 3360.00, 1681.93, 1608.63, 1024.20 cm^−1^; ^1^H and ^13^C (DMSO-*d*_6_ in 700 MHz and 175 MHz) spectral data, see Table 1; Mass spectrum (HRESIMS) *m*/*z* 371.1153 [M − H]^−^ (calcd for C_21_H_16_N_4_O_3_, 371.1150).

Fumigatoside F (**2**)*.* White amorphous solid; UV (MeOH) λ_max_ (log ε): 210 (0.78), 230 (0.58), 250 (0.21) nm; 3354.21, 1662.64, 1653.00, 1606.70, 1473.62, 1373.32 cm^−1^; ^1^H and ^13^C (DMSO-*d*_6_ in 700 MHz and 175 MHz) spectral data, see Table 1; Mass spectrum (HRESIMS) *m*/*z* 419.1364 [M − H]^−^ (calcd for C_22_H_19_N4O_5_, 419.1361).

3,7-diketo-cephalosporin P_1_ (**6**)*.* White amorphous solid; UV (MeOH) λ_max_ (log ε): 202 (1.00), 220 (0.5), 254 (0.3) nm; IR (KBr) *V*_max_: 3398.57, 1705.07, 1645.28, 1463.97, 1249.87, 1024.20 cm^−1^; ^1^H and ^13^C (DMSO-*d*_6_ in 700 MHz and 175 MHz) spectral data, see Table 2; Mass spectrum (HRESIMS) *m*/*z* 551.2968 [M + Na]^+^ (calcd for C_31_H_44_O_7_, 551.2979).

### 3.5. Biological Activity

In vitro antimicrobial activity was evaluated by using the Kirby-Bauer broth micro dilution method, which was previously described [25]. Antibacterial activity was evaluated against *S. aureus* (ATCC 16339, 29213), *A. baumanii* (ATCC 19606, 15122) and *K. pneumonia* (ATCC 14578), while antifungal activity was evaluated against *Fusarium oxysporum* f.sp. *cucumerinu* and *Fusarium oxysporum* f. sp. *momordicae.* The bacterial pathogens were cultivated on LB agar plates at 37 °C for 24 h, for fungi on potato dextrose agar (PDA) media at 28 °C for 3 days and test compounds dissolved in DMSO at different concentrations from 50 to 0.03 µg/mL. Streptomycin was used as the positive control for bacteria and nystatin was used for fungal pathogens in the minimum inhibitory concentration (MIC) test.

## 4. Conclusions

The chemical investigation of the deep-sea-derived fungus *Aspergillus fumigatus* SCSIO 41012 has led to eight compounds, including three new metabolites. Their structures were elucidated by the detailed analysis of spectroscopic data. Compounds **1**–**4** are quinazoline-containing indole alkaloids, which are widely produced by filamentous fungi, particularly *A. fumigatus*. In our previous investigations, three new quinazoline glucosides, namely, fumigatosides B–D, were isolated from fungus *A. fumigatus* derived from a jellyfish. However, none of the three compounds exhibited antibacterial activity. In this study, compounds **1**–**8** were also evaluated for their antibacterial and antifungal inhibitory activities, among which compound **1** has comparable or even higher antibacterial activity than other indole alkaloids. Compound **1** also showed significant antifungal activity against *Fusarium oxysporum* f. sp. *momordicae* with MIC at 1.5 µg/mL. Compound **4** exhibited significant higher activity against *S. aureus* (16339 and 29213) with MIC values of 1.565, and 0.78 µg/mL, respectively, and compound **2** exhibited significant activity against *A. baumanii* ATCC 19606 with MIC value of 6.25 µg/mL.

## Figures and Tables

**Table 1:** ^1^H (700 MHz) and ^13^C (175 MHz) NMR and HMBC data for **1** (DMSO-*d*_6_) and **2** (DMSO-*d*_6_).

Position	1		2	
	*δ*_H_ (*J* in Hz)	*δ*_C_ Type	*δ*_H_ (*J* in Hz)	*δ*_C_ Type
1		169.4 qC		
2			5.52 s	82.8 CH
3		58.4 qC		90.9 qC
4		153.3 qC		131.4 qC
5			7.84 d (10.5)	126.1 CH
6		147.0 qC	7.35 m	126.3 CH
7	7.67 d (8.4)	127.3 CH	7.49 m	131.4 CH
8	7.82 td (8.4, 1.4)	135.3 CH	8.47 m	115.1 CH
9	7.54 td (8.4, 1.4)	127.9 CH		138.5 qC
10	8.16 dd (8.4, 1.4)	126.8 CH		
11		120.7 qC		172.2 C
12		159.8 qC	5.50 t (14.0)	57.4 CH
13			3.49 dd (14.0, 18.2)	32.7 CH_2_
			2.62 dd (14.0, 18.2)	
14	5.69 t (17.5, 2.8)	54.3 CH		172.6 qC
15	3.42 dd (17.5, 2.8)	26.1 CH_2_	4.26 t (9.1)	58.3 CH
	3.24 dd (17.5, 4.9)			
16		106.5 qC		
17		127.3 qC		
18	7.41 d (7.0)	118.5 CH		160.2 qC
19	6.99 t (7.0)	119.7 CH		121.8 qC
20	7.12 td (7.0, 0.7)	122.7 CH	8.25 d (10.5)	126.8 CH
21	7.40 d (7.0)	112.4 CH	7.65 t (9.1)	128.1 CH
22		132.5	7.78 t (9.1)	135.5 CH
23			7.76 m	127.9 CH
24		135.7		148.1 qC
25	4.65 dd (25.2, 11.9)	60.4		
26			8.54 s	148.1 CH
27			1.25 d (9.8)	18.8 CH_3_

**Table 2:** The ^1^H and ^13^C data of compound **6** (DMSO-*d*_6_, 700 MHz and 175 MHz in *δ* ppm).

Position	*δ*_H_ (*J* in Hz)	*δ*_C_ Type	Position	*δ*_H_ (*J* in Hz)	*δ*_C_ Type
1	2.54 m	37.2 CH_2_	16	5.65 d (8.4)	73.8 CH
	1.69 m		17		145.1 qC
2	1.99 m	33.0 CH_2_	18	0.84 s	18.6 CH_3_
	1.48 m		19	1.27 s	24.0 CH_3_
3		213.6 qC	20		131.4 qC
4	2.54 dd (12.6, 6.5)	40.7 CH	21		171.5 qC
5	2.61 d (12.6)	45.1 CH	22	2.45 m	28.7 CH_2_
6	3.71 brs	73.2 CH	23	2.08 m	23.4 CH_2_
7		214.7 qC		2.04 m	
8		52.4 qC	24	5.10 t (7.5)	123.7 CH
9	2.66 dd (13.2, 2.5)	41.5 CH	25		132.0 qC
10		35.0 qC	26	1.56 s	17.1 CH_3_
11	1.99 m	22.6 CH_2_	27	1.65 s	26.0 CH_3_
	1.75 m		28	0.98 d (7.0)	12.8 CH_3_
12	1.82 dd (12.8, 3.6)	26.1 CH_2_	29	1.18 s	18.0 CH_3_
13	2.58 d (11.2)	48.4 CH	1′		170.2 qC
14		46.6 qC	2′	1.89 s	20.9 CH_3_
15	2.87 d (14.5)	40.9 CH_2_			
	2.20 dd (14.5,8.5)				

**Table 3:** Minimum inhibitory concentration of purified compounds from *Aspergillus fumigatus* SCSIO 41012.

Compounds	19606	15122	16339	29213	14578	Fungal Isolate 1	Fungal Isolate 2
1	12.5 ± 0.042	6.25 ± 0.035	6.25 ± 0.13	---	12.5 ± 0.098	25 ± 0.04	1.565 ± 0.098
2	6.25 ± 0.033	---	---	---	---	---	---
							
3	---	---	1.565 ± 0.04	0.78 ± 0.025	25 ± 0.05	12.5 ± 0.084	---
4	---	6.25 ± 0.083	12.5 ± 0.33	12.5 ± 0.018	25 ± 0.003	25 ± 0.071	---
5	50 ± 0.074	6.25 ± 0.09	---	---	---	12.5 ± 0.09	---
6	50 ± 0.020	---	---	---	---	---	---
7	---	---	25 ± 0.082	12.5 ± 0.050	---	---	---
8	---	12.5 ± 0.045	---	---	3.125 ± 0.08	1.565 ± 0.07	---
Streptomycin	1.565 ± 0.04	12.5 ± 0.078	6.25 ± 0.04	3.125 ± 0.11	0.78 ± 0.18	---	---
Nystatin	---	---	---	---	---	3.125 ± 0.034	12.5 ± 0.02

## Supplementary Materials

The following are available online. Figure S1: ^1^H NMR spectra (700 MHz, DMSO-*d*_6_) of the new compound 1, Figure S2: ^13^C NMR spectra (175 MHz, DMSO-*d*_6_) of the new compound 1, Figure S3: ^13^C DEPT spectra of the new compound 1, Figure S4: ^1^H-^1^H COSY spectra of the new compound 1, Figure S5: HMBC spectra of the new compound 1, Figure S6: HMQC spectra of the new compound 1, Figure S7: IR spectra of the new compound 1, Figure S8: HRESIMS of the new compound 1, Figure S9: The experimental CD curve of the new compound 1, Figure S10: ^1^H NMR spectra (700 MHz, DMSO-*d*_6_) of the new compound 2, Figure S11: ^13^C NMR spectra (175 MHz, DMSO-*d*_6_) of the new compound 2, Figure S12: ^13^C DEPT spectra of the new compound 2, Figure S13: ^1^H-^1^H COSY spectra of the new compound 2, Figure S14: HMQC spectra of the new compound 2, Figure S15: HMBC spectra of the new compound 2, Figure S16: NOESY spectra of the new compound 2, Figure S17: IR spectra of the new compound 2, Figure S18: HRESIMS of the new compound 2, Figure S19: The experimental CD curve of the new compound 2, Figure S20: 1H NMR spectra (700 MHz, DMSO-*d*_6_) of the new compound 3, Figure S21: ^13^C NMR spectra (175 MHz, DMSO-*d*_6_) of the new compound 3, Figure S22: ^13^C DEPT spectra of the new compound 3, Figure S23: ^1^H-^1^H COSY spectra of the new compound 3, Figure S24: HMQC spectra of the new compound 3, Figure S25: HMBC spectra of the new compound 3, Figure S26: NOESY spectra of the new compound 3, Figure S27: IR spectra of the new compound 3, Figure S28: HRESIMS of the new compound 3, Figure S29: The experimental CD curve of the new compound 3.
