# Supplementary material for: Bioactive Novel Indole Alkaloids and Steroids from Deep Sea-Derived Fungus Aspergillus fumigatus SCSIO 41012
Source: Molecules. 2018 Sep 18;23(9):2379. doi: 10.3390/molecules23092379 (PMC6225233; doi:10.3390/molecules23092379)
Supplement: Supplementary file 1 [file molecules-23-02379-s001.pdf]

Bioactive Novel Indole Alkaloids and Steroids from Deep Sea-Derived Fungus *Aspergillus fumigatus* SCSIO 41012

Salendra Limbadri [1,2], Xiaowei Luo [1,2], Xiuping Lin [1], Shengrong Liao [1], Junfeng Wang [1], Xuefeng Zhou [1], Bin Yang [1,*] and Yonghong Liu [1,*]

[1] Key Laboratory of Tropical Marine Bio-resources and Ecology/Guangdong Key Laboratory of Marine Materia Medica/Research Center for Marine Microbes, South China Sea Institute of Oceanology, Chinese Academy of Sciences, Guangzhou 510301, China;
E-Mails: raj.badri202@gmail.com (S.L.); luoxiaowei14@mails.ucas.ac.cn (X.L.); xiupinglin@hotmail.com (X.L.); ljrss@126.com (S.L.); junfeng1982a@163.com (J.W.); xfzhou@scsio.ac.cn (X.Z.);

[2] University of Chinese Academy of Sciences, Beijing, China

* Author to whom correspondence should be addressed; E-Mail: bingo525@163.com (B.Y.); yonghongliu@scsio.ac.cn (Y.L.); Tel./Fax: +86-020-8902-3244.

Academic Editor: name
Received: date; Accepted: date; Published: date

Abstract: Two new alkaloids, fumigatosides E (**1**) and F (**2**), and a new natural product, 3, 7-diketo-cephalosporin P_1 (**6**) along with five known compounds (**3**–**5**, **7**, **8**) were isolated from deep-sea derived fungal *Aspergillus fumigatus* SCSIO 41012. Their structures were determined by extensive spectroscopic data analysis, including 1D, 2D NMR and MS, and comparison between the calculated and experimental ECD spectra. In addition, all compounds were tested for antibacterial and antifungal inhibitory activities. Compound **1** showed significant antifungal activity against *Fusarium oxysporum* f. sp. *momordicae* with MIC at 1.56 μg/mL. Compound **4** exhibited significant higher activity against *S. aureus* (16339 and 29213) with MIC values of 1.56, and 0.78 μg/mL, respectively, and compound **2** exhibited significant activity against *A. baumanii* ATCC 19606 with MIC value of 6.25 μg/mL.

Keywords: deep sea-derived fungus; *Aspergillus fumigatus* SCSIO 41012; indole alkaloids; steroids; antibacterial activity; antifungal activity

List of supporting information

Figure S1: ^{1}H NMR spectra (700 MHz, DMSO-*d6*) of the new compound **1**.

Figure S2: ^{13}C NMR spectra (175 MHz, DMSO-*d6*) of the new compound **1**.

Figure S3: ^{13}C DEPT spectra of the new compound **1**.

Figure S4: ^{1}H-^{1}H COSY spectra of the new compound **1**.

Figure S5: HMBC spectra of the new compound **1**.

Figure S6: HMQC spectra of the new compound **1**.

Figure S7: IR spectra of the new compound **1**.

Figure S8: HRESIMS of the new compound **1**.

Figure S9: The experimental CD curve of the new compound **1**.

Figure S10: ^{1}H NMR spectra (700 MHz, DMSO-*d6*) of the new compound **2**.

Figure S11: ^{13}C NMR spectra (175 MHz, DMSO-*d6*) of the new compound **2**.

Figure S12: ^{13}C DEPT spectra of the new compound **2**.

Figure S13: ^{1}H-^{1}H COSY spectra of the new compound **2**.

Figure S14: HMQC spectra of the new compound **2**.

Figure S15: HMBC spectra of the new compound **2**.

Figure S16: NOESY spectra of the new compound **2.**

Figure S17: IR spectra of the new compound **2**.

Figure S18: HRESIMS of the new compound **2**.

Figure S19: The experimental CD curve of the new compound **2**.

Figure S20: ^{1}H NMR spectra (700 MHz, DMSO-*d6*) of the new compound **3**.

Figure S21: ^{13}C NMR spectra (175 MHz, DMSO-*d6*) of the new compound **3**.

Figure S22: ^{13}C DEPT spectra of the new compound **3**.

Figure S23: ^{1}H-^{1}H COSY spectra of the new compound **3**.

Figure S24: HMQC spectra of the new compound **3**.

Figure S25: HMBC spectra of the new compound **3**.

Figure S26: NOESY spectra of the new compound **3.**

Figure S27: IR spectra of the new compound **3**.

Figure S28: HRESIMS of the new compound **3**.

Figure S29: The experimental CD curve of the new compound **3**.

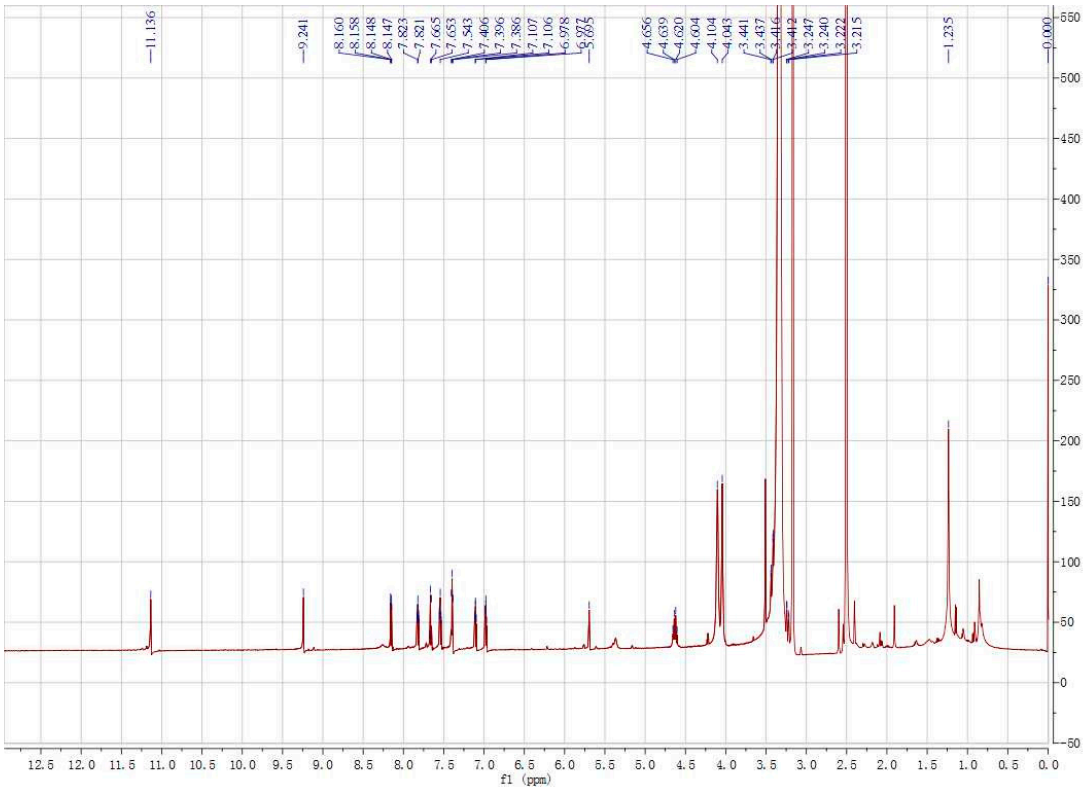

Figure S1. ^{1}H NMR spectra (700 MHZ, DMSO-*d6*) of the new compound **1**.

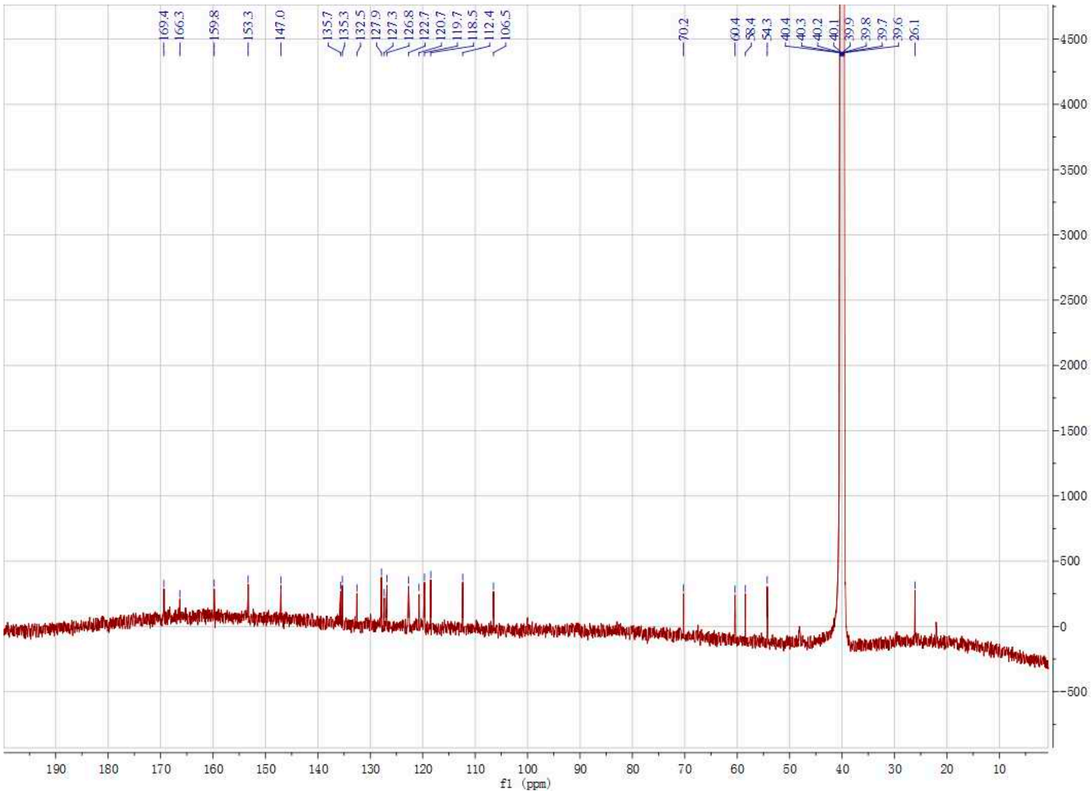

Figure S2. ^{13}C NMR spectra (175 MHZ, DMSO-*d6*) of the new compound **1.**

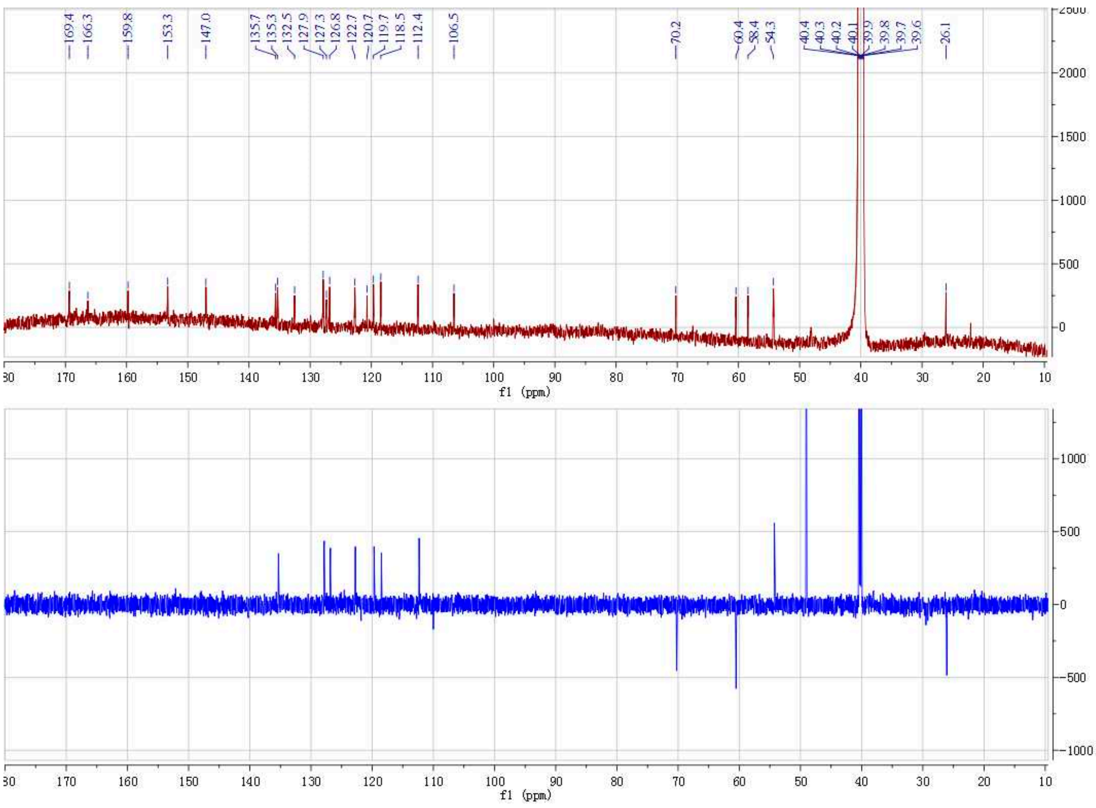

Figure S3. DEPT spectra of the new compound **1.**

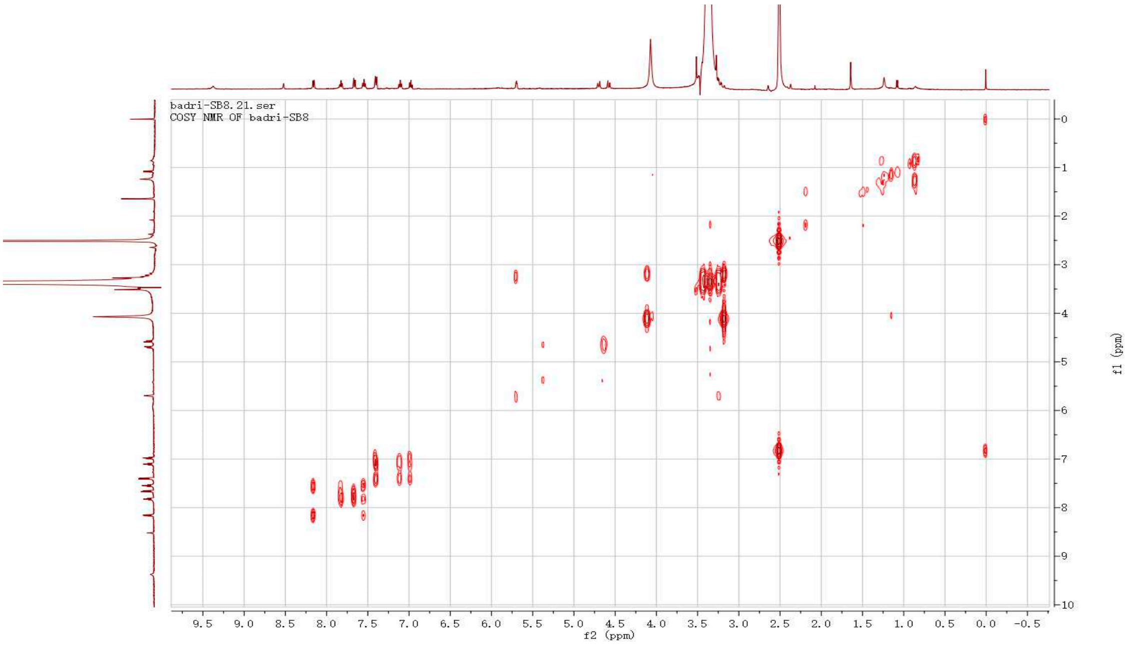

Figure S4. COSY spectra of the new compound **1**.

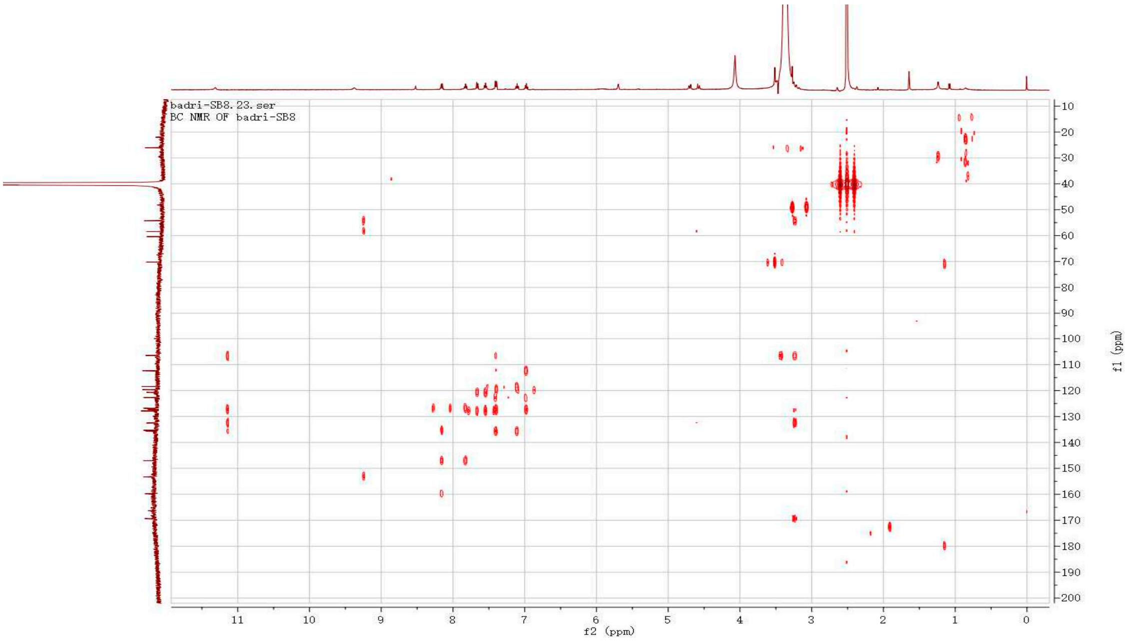

Figure S5. HMBC spectra of the new compound **1.**

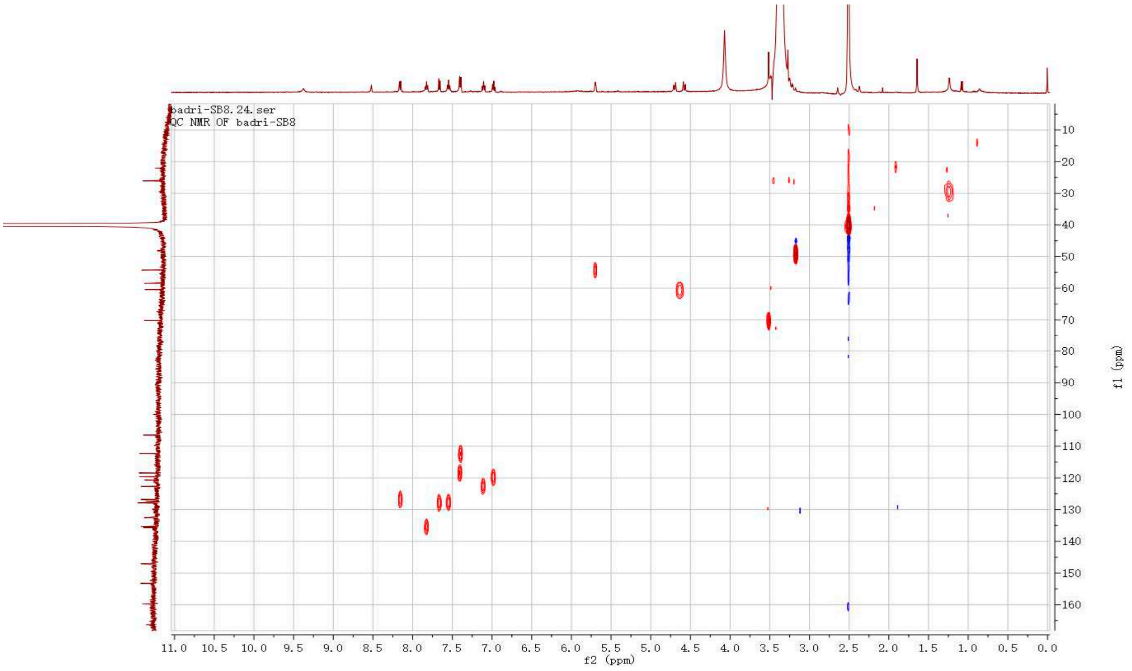

Figure S6. HMQC spectra of the new compound **1**.

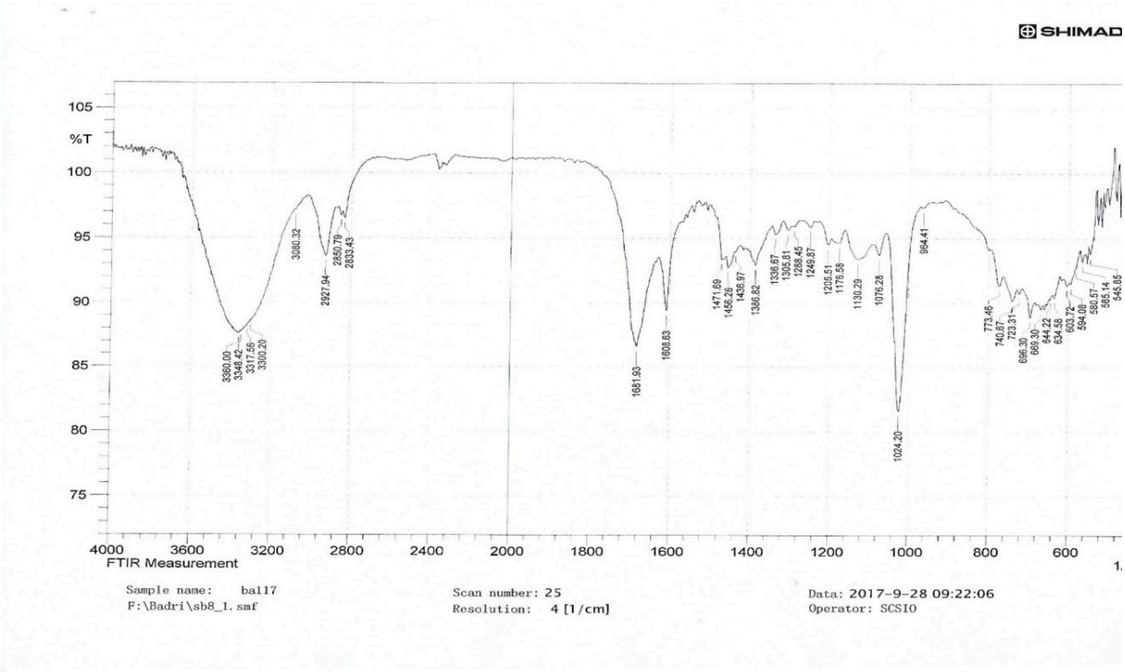

Figure S7. IR spectra of the new compound **1**.

Mass Spectrum SmartFormula Report

Analysis Info

Analysis Name	D:\Data\MS\data\201707\BADRI_SB8_neg_41_01_3244.d	Acquisition Date	7/17/2017 9:25:51 AM
Method	LC_Direct Infusion_neg_100-1000mz.m	Operator	SCSIO
Sample Name	BADRI_SB8_neg	Instrument	maXis 255552.00029
Comment			

Acquisition Parameter

Source Type	ESI	Ion Polarity	Negative	Set Nebulizer	0.4 Bar
Focus	Active	Set Capillary	4000 V	Set Dry Heater	180 °C
Scan Begin	100 m/z	Set End Plate Offset	-500 V	Set Dry Gas	4.0 l/min
Scan End	2000 m/z	Set Charging Voltage	0 V	Set Divert Valve	Waste
		Set Corona	0 nA	Set APCI Heater	0 °C

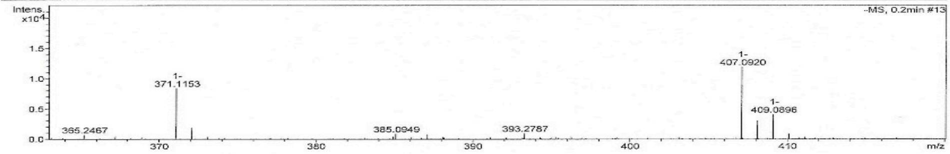

Meas. m/z	#	Ion Formula	Score	m/z	err [ppm]	err [mDa]	mSigma	rdb	e⁻ Conf	N-Rule
371.115323	1	C20H19O7	52.06	371.113627	-4.6	-1.7	4.5	11.5	even	ok
	2	C21H15N4O3	100.00	371.114964	1.0	0.4	11.2	16.5	even	ok
407.091999	1	C21H16ClN4O3	100.00	407.091642	-0.9	-0.4	9.0	15.5	even	ok
	2	C20H20ClO7	39.62	407.090304	4.2	1.7	16.9	10.5	even	ok
779.213223	1	C40H40ClO14	42.44	779.211207	-2.6	-2.0	20.5	20.5	even	ok
	2	C42H32ClN8O6	92.37	779.213682	0.8	0.7	24.3	30.5	even	ok

BADRI_SB8_neg_41_01_3244.d
Bruker Compass DataAnalysis 4.1 printed: 7/17/2017 10:49:49 AM by: SCSIO Page 1 of 1

Figure S8. HRESIMS of the new compound **1**.

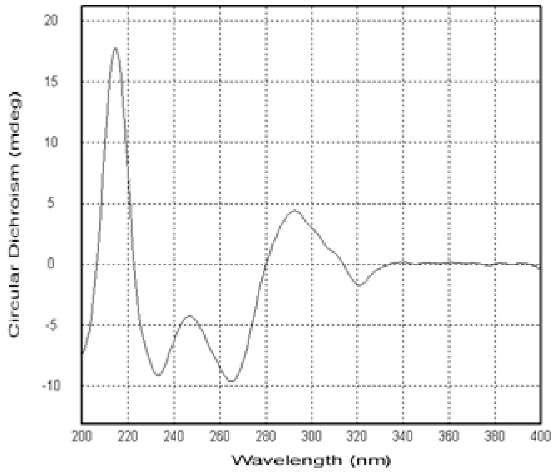

Figure S9. The experimental CD curve of the new compound **1.**

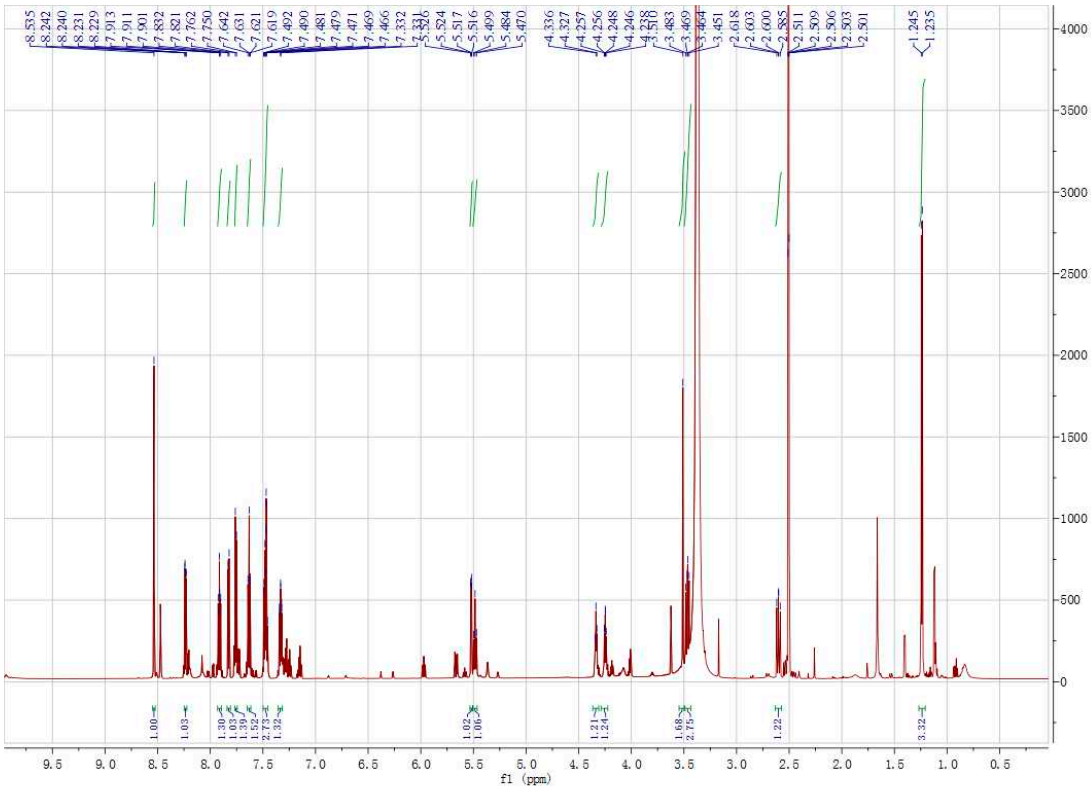

Figure S10. ¹H NMR spectra (700 MHZ, DMSO-*d6*) of the new compound **2**.

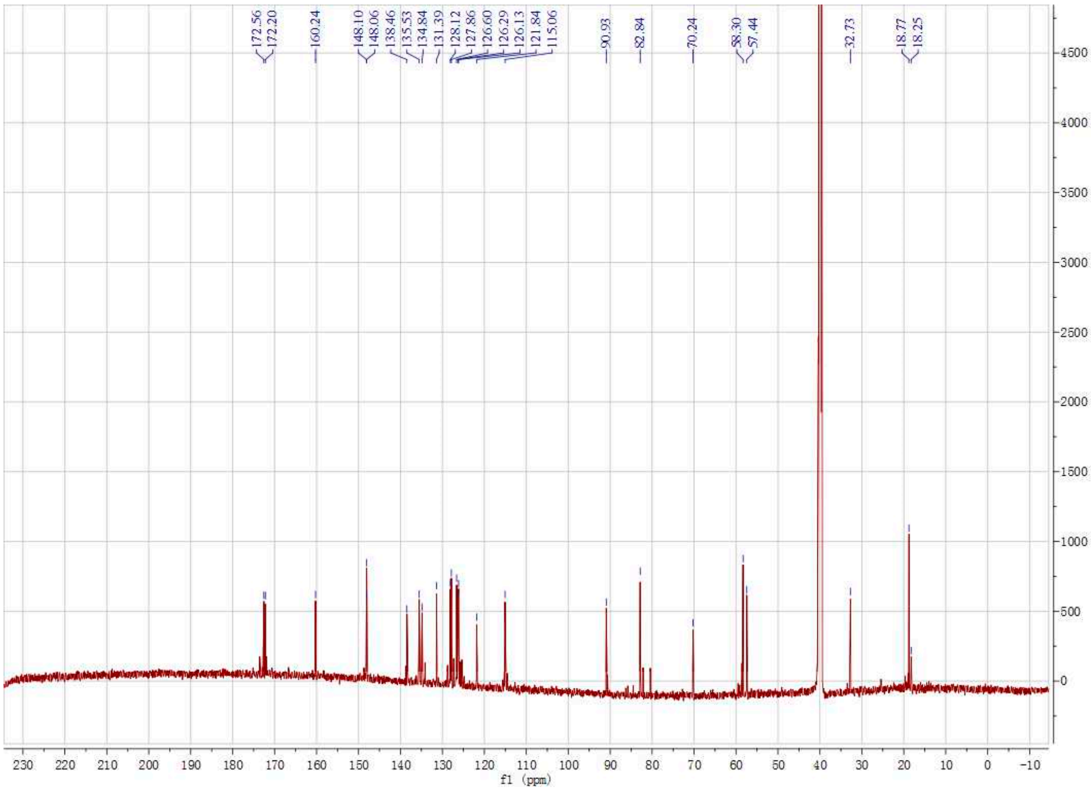

Figure S11. ^{13}C NMR spectra (175 MHZ, DMSO-*d6*) of the new compound **2.**

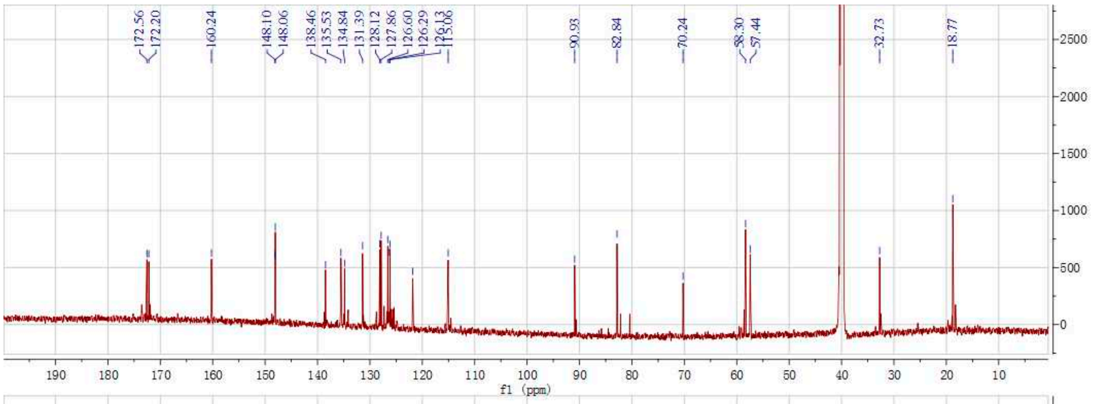

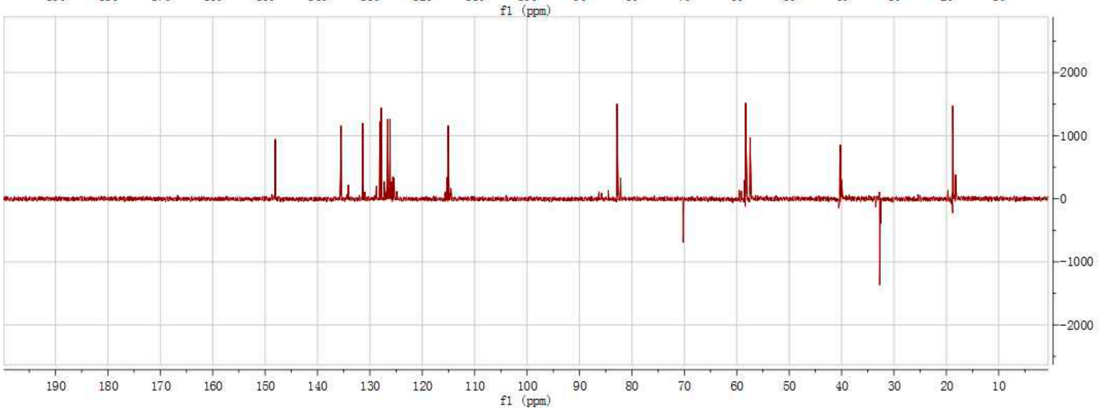

Figure S12. DEPT spectra of the new compound **2.**

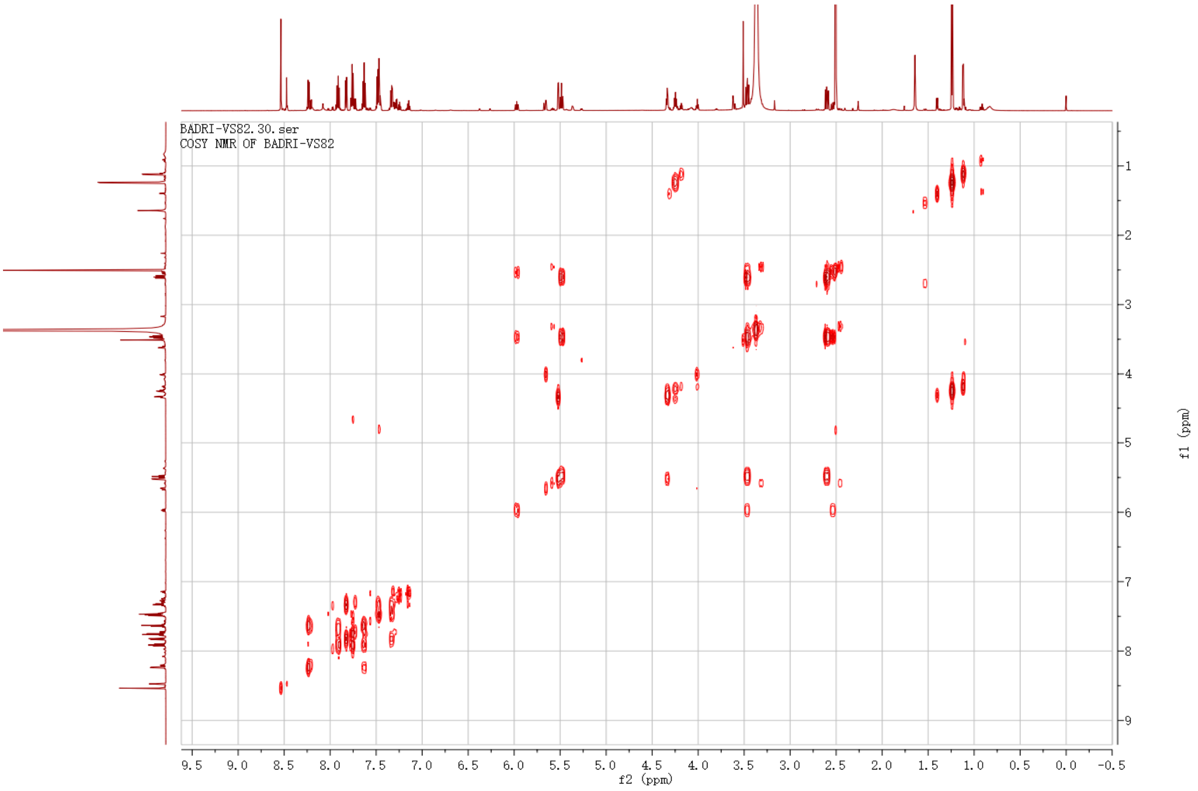

Figure S13. COSY spectra of the new compound **2**.

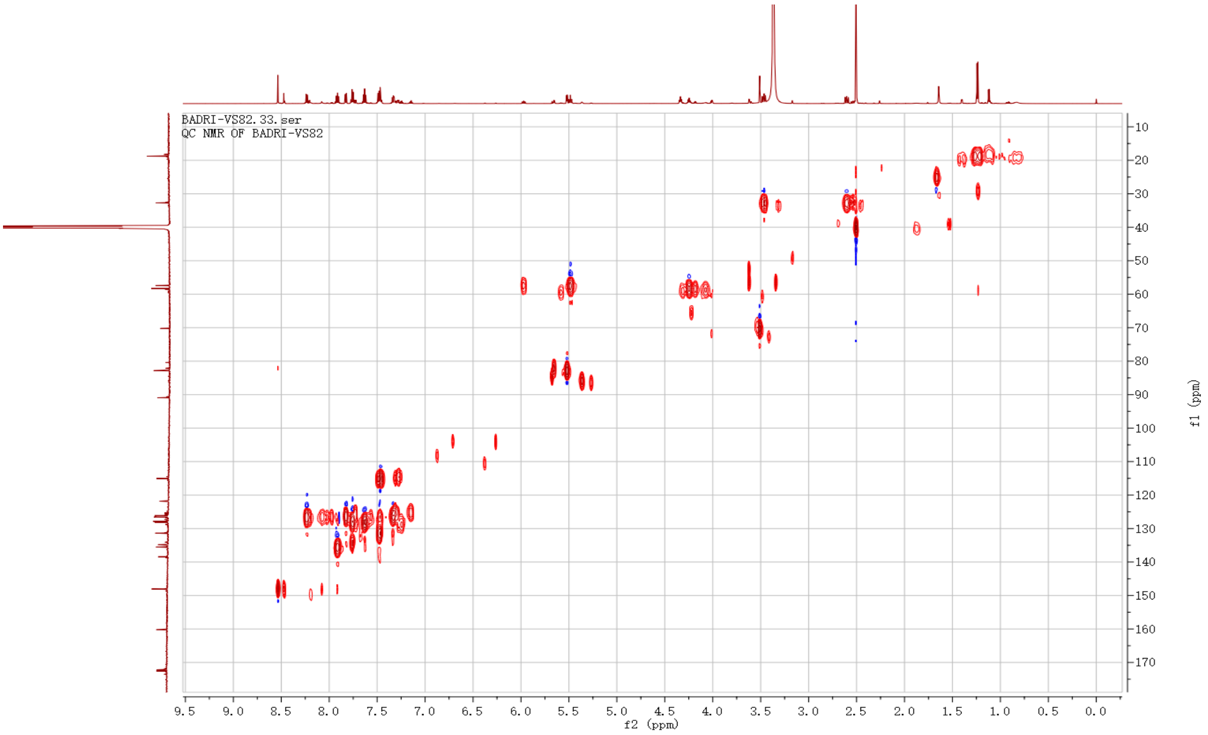

Figure S14. HMQC spectra of the new compound **2**.

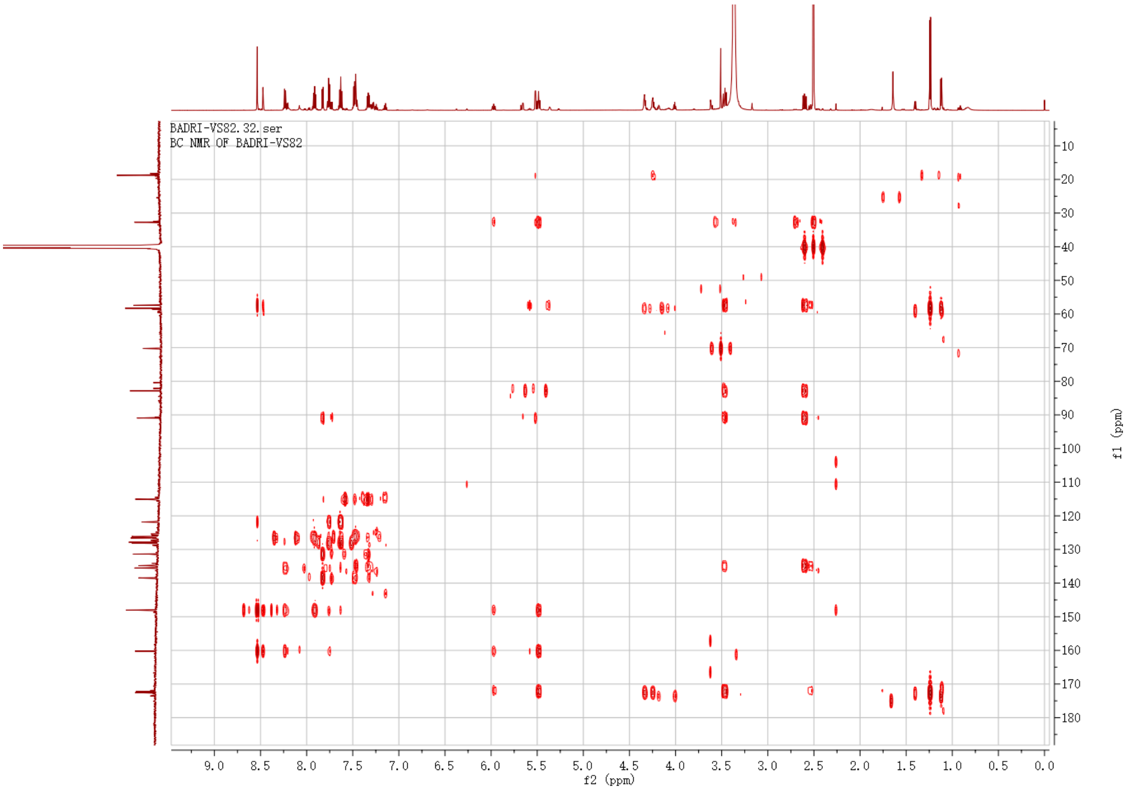

Figure S15. HMBC spectra of the new compound **2.**

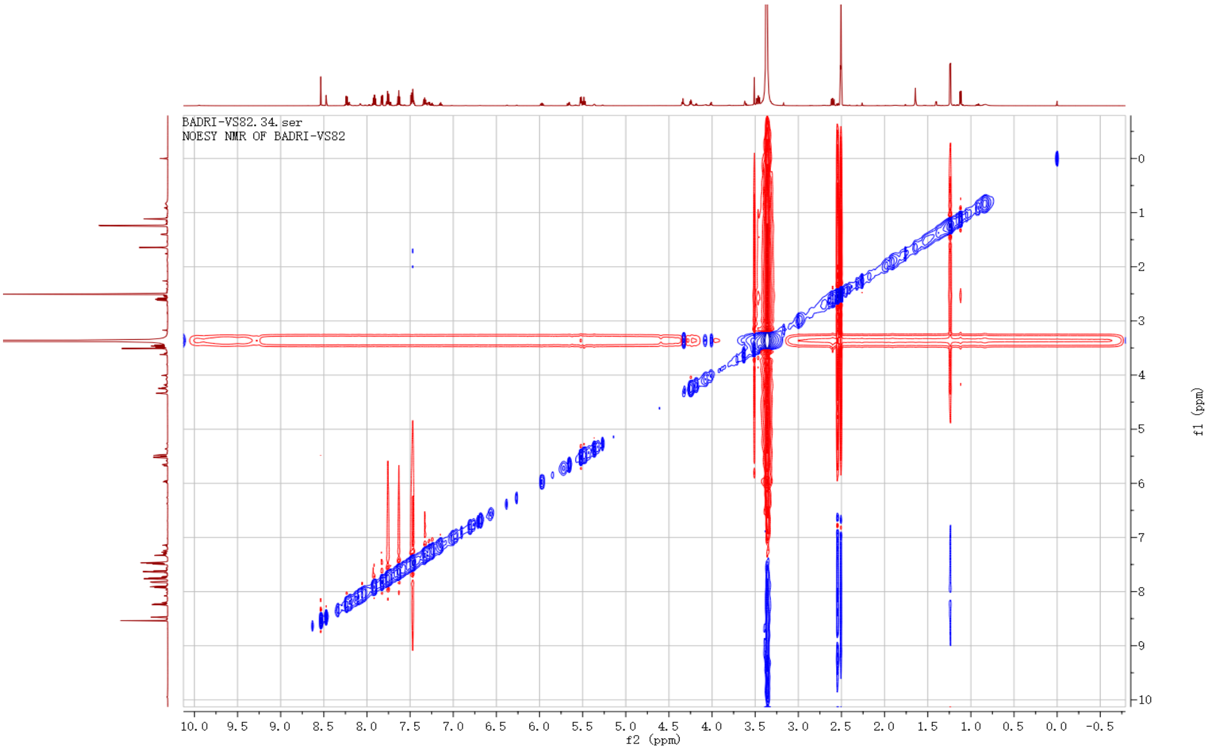

Figure S16. NOESY spectra of the new compound **2.**

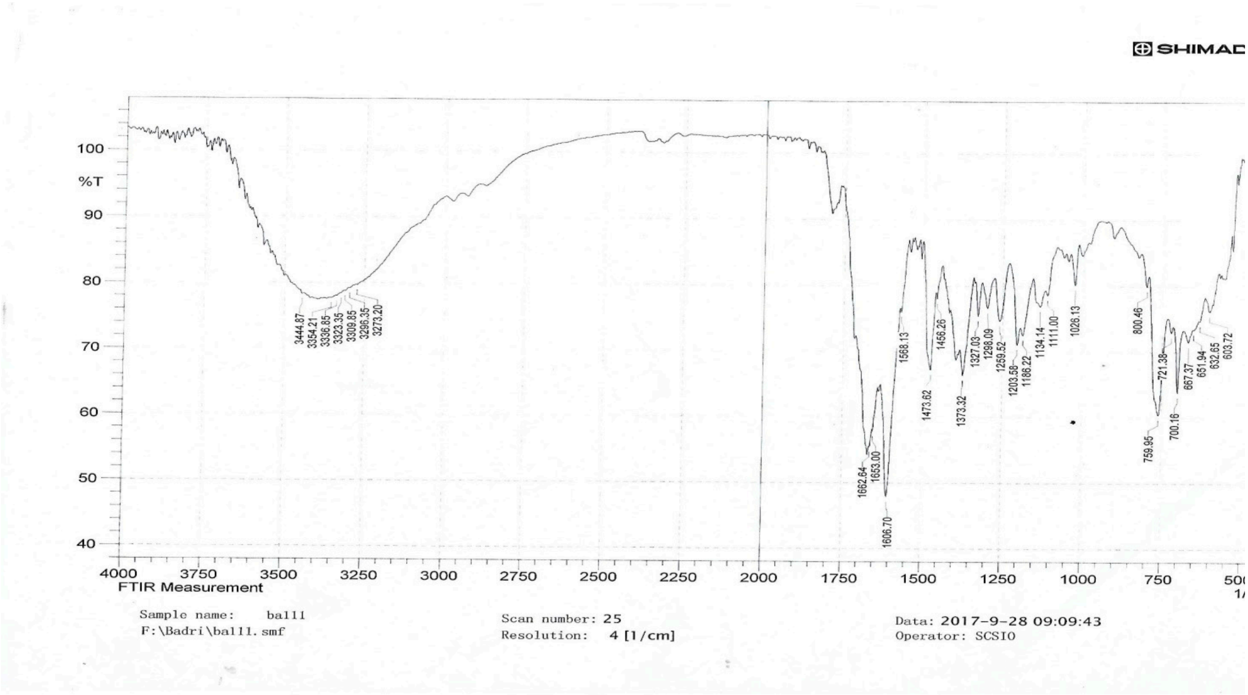

Figure S17. IR spectra of the new compound **2**.

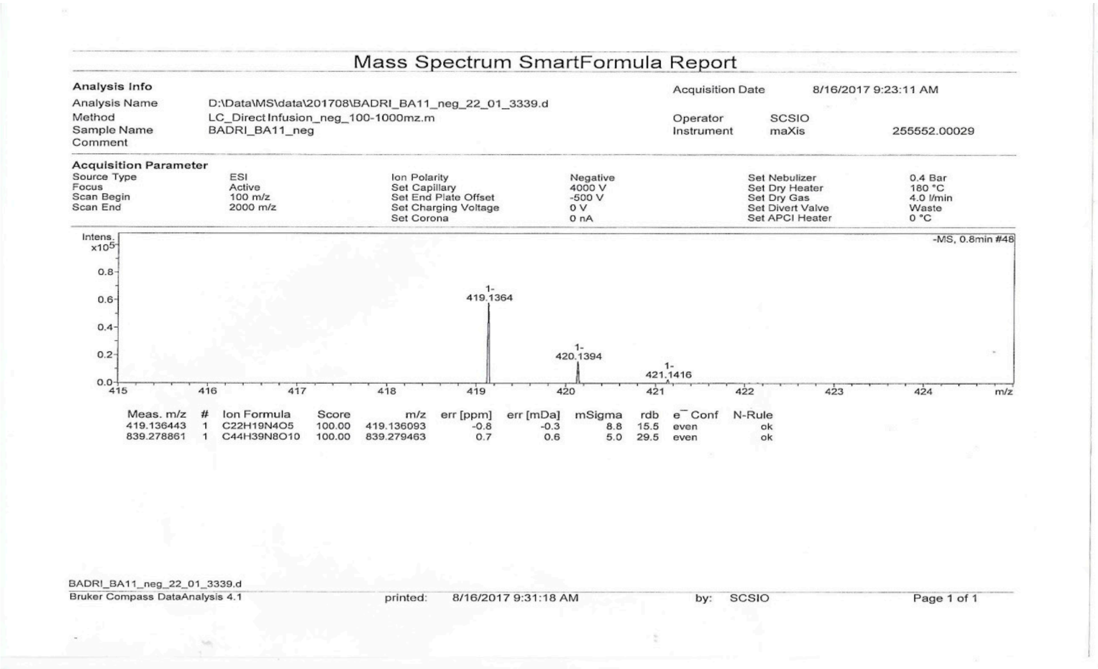

Mass Spectrum SmartFormula Report

Analysis Info				
Analysis Name	D:\Data\MS\data\201708\BADRI_BA11_neg_22_01_3339.d	Acquisition Date	8/16/2017 9:23:11 AM	
Method	LC_Direct Infusion_neg_100-1000mz.m	Operator	SCSIO	
Sample Name	BADRI_BA11_neg	Instrument	maXis	255552.00029
Comment				

Acquisition Parameter					
Source Type	ESI	Ion Polarity	Negative	Set Nebulizer	0.4 Bar
Focus	Active	Set Capillary	4000 V	Set Dry Heater	180 °C
Scan Begin	100 m/z	Set End Plate Offset	-500 V	Set Dry Gas	4.0 l/min
Scan End	2000 m/z	Set Charging Voltage	0 V	Set Divert Valve	Waste
		Set Corona	0 nA	Set APCI Heater	0 °C

Meas. m/z	#	Ion Formula	Score	m/z	err [ppm]	err [mDa]	mSigma	rdb	e⁻ Conf	N-Rule
419.136443	1	C22H19N4O5	100.00	419.136093	-0.8	-0.3	8.8	15.5	even	ok
839.278861	1	C44H39N8O10	100.00	839.279463	0.7	0.6	5.0	29.5	even	ok

BADRI_BA11_neg_22_01_3339.d
Bruker Compass DataAnalysis 4.1 printed: 8/16/2017 9:31:18 AM by: SCSIO Page 1 of 1

Figure S18. HRESIMS of the new compound **2**.

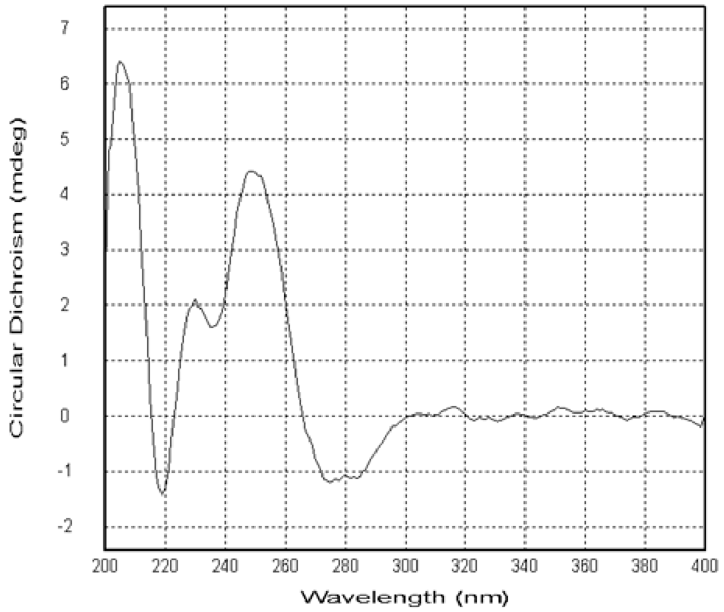

Figure S19. The experimental CD curve of the new compound **2.**

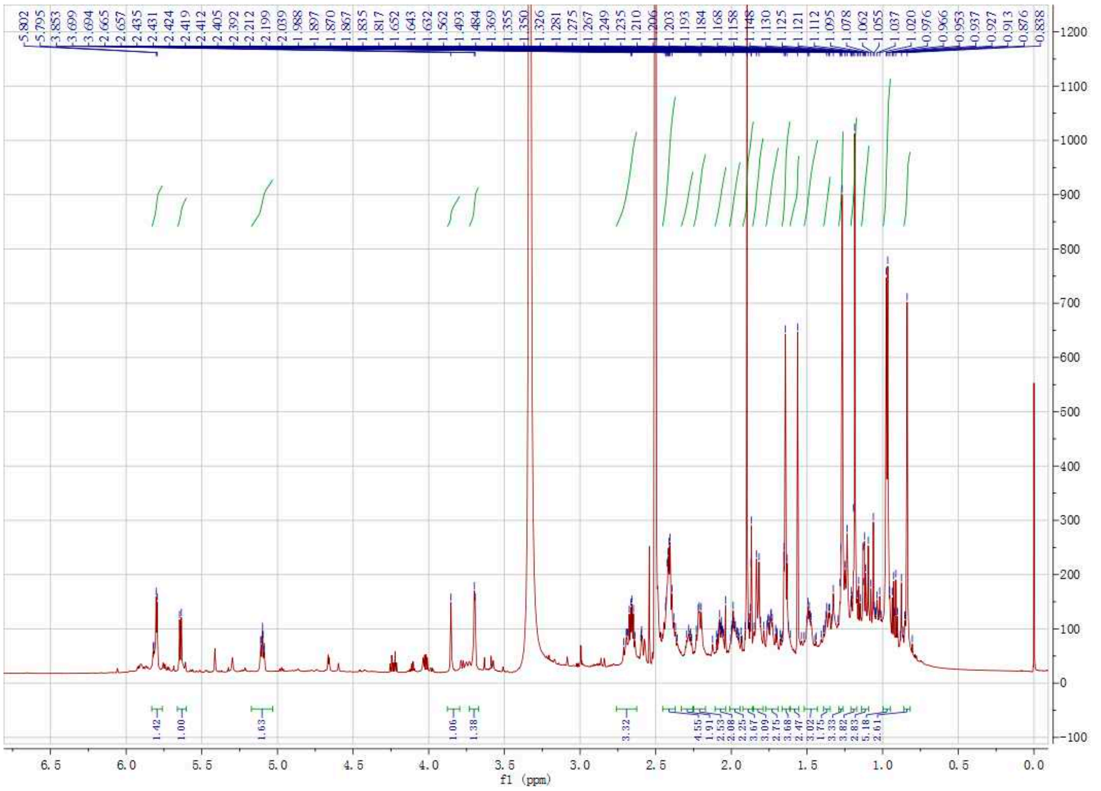

Figure S20. ^{1}H NMR spectra (700 MHZ, DMSO-*d6*) of the new compound **3.**

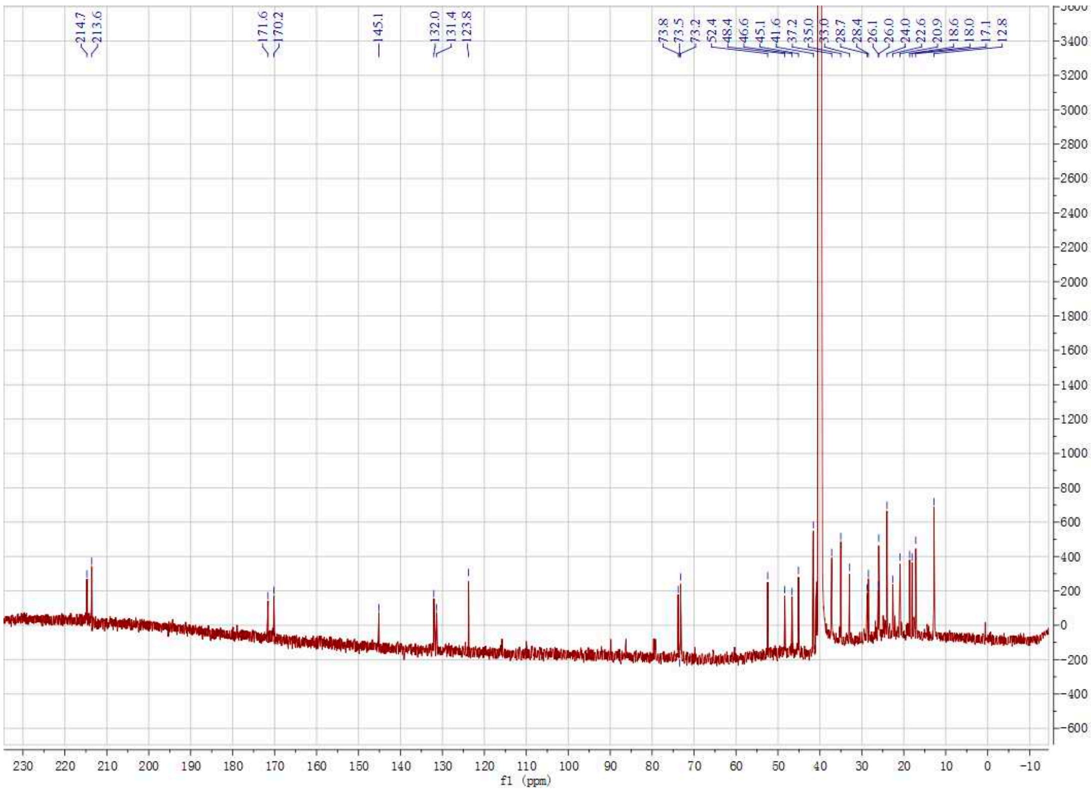

Figure S21. ^{13}C NMR spectra (175 MHZ, DMSO-*d6*) of the new compound **3.**

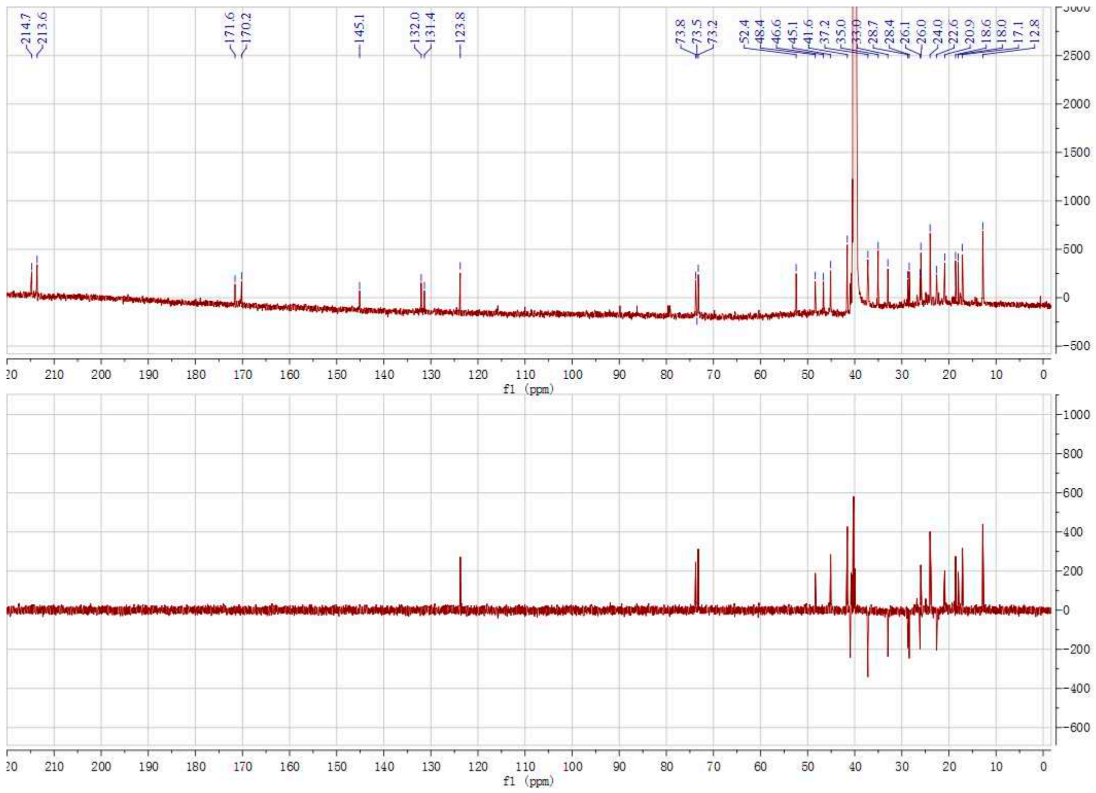

Figure S22. DEPT spectra of the new compound **3.**

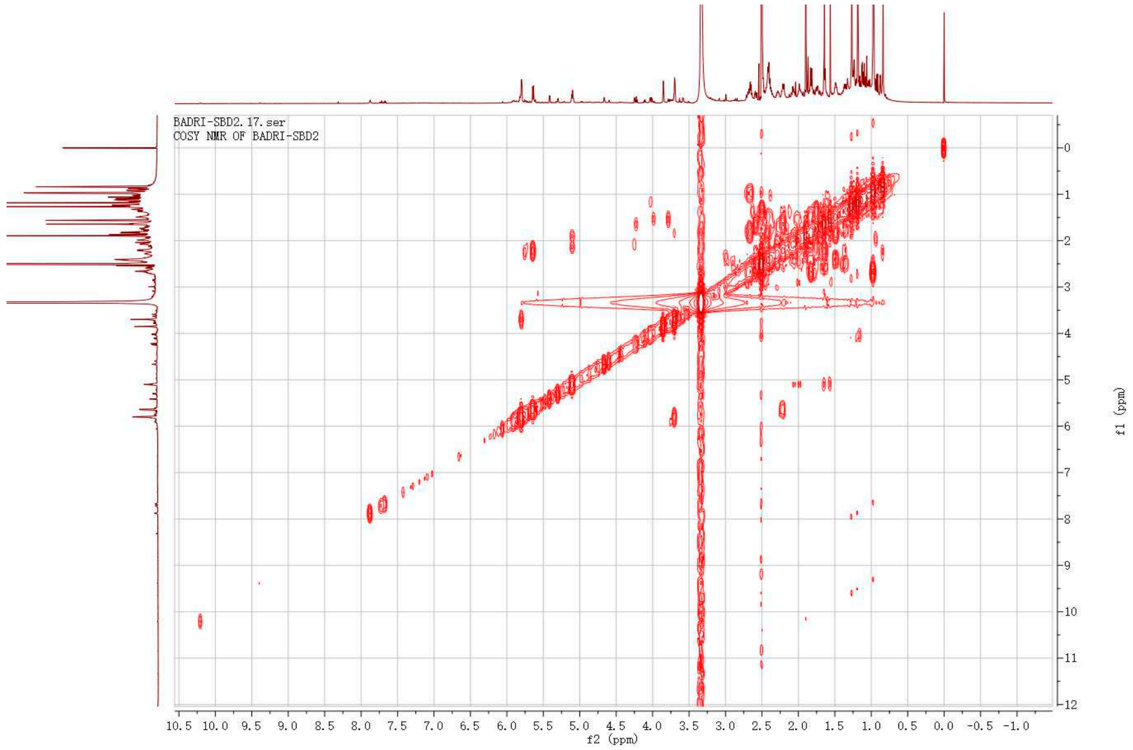

Figure S23. COSY spectra of the new compound **3.**

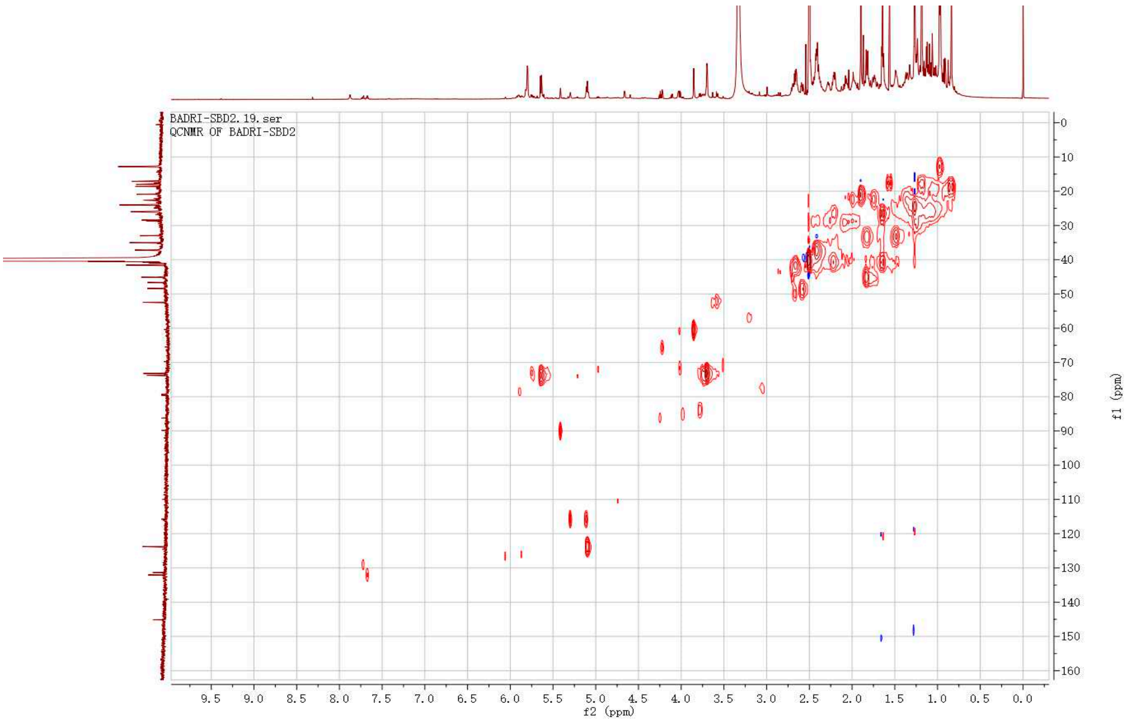

Figure S24. HMQC spectra of the new compound **3.**

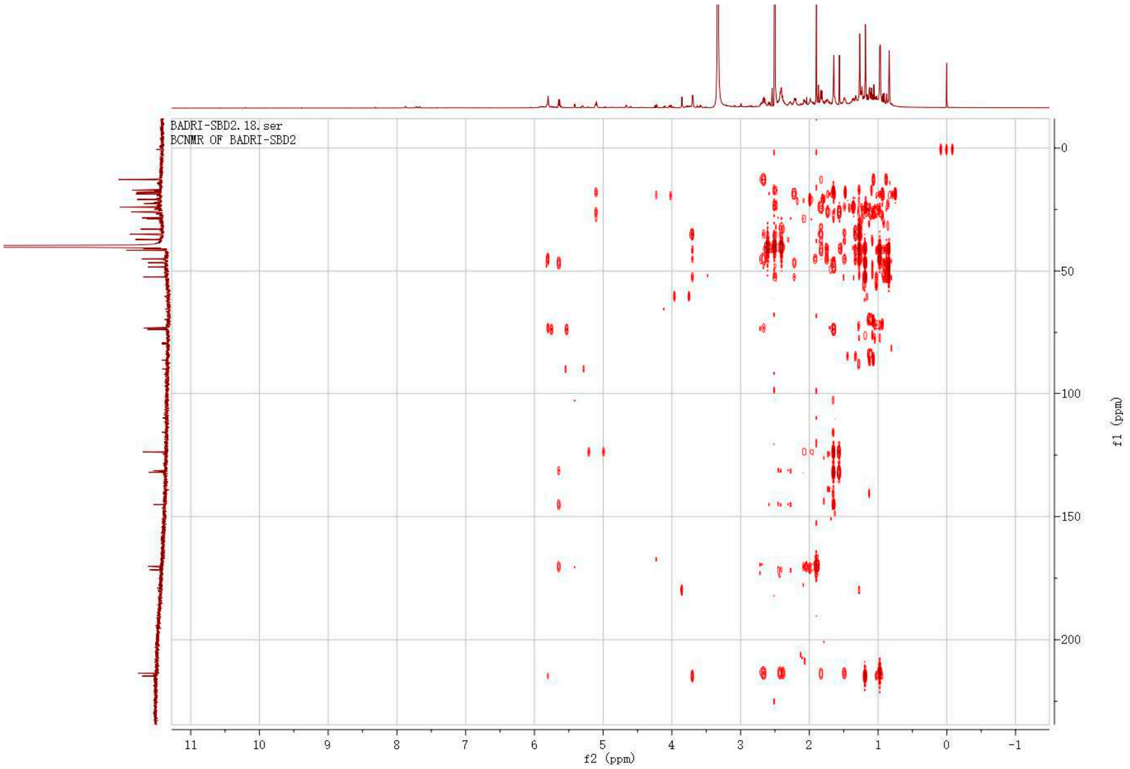

Figure S25. HMBC spectra of the new compound **3.**

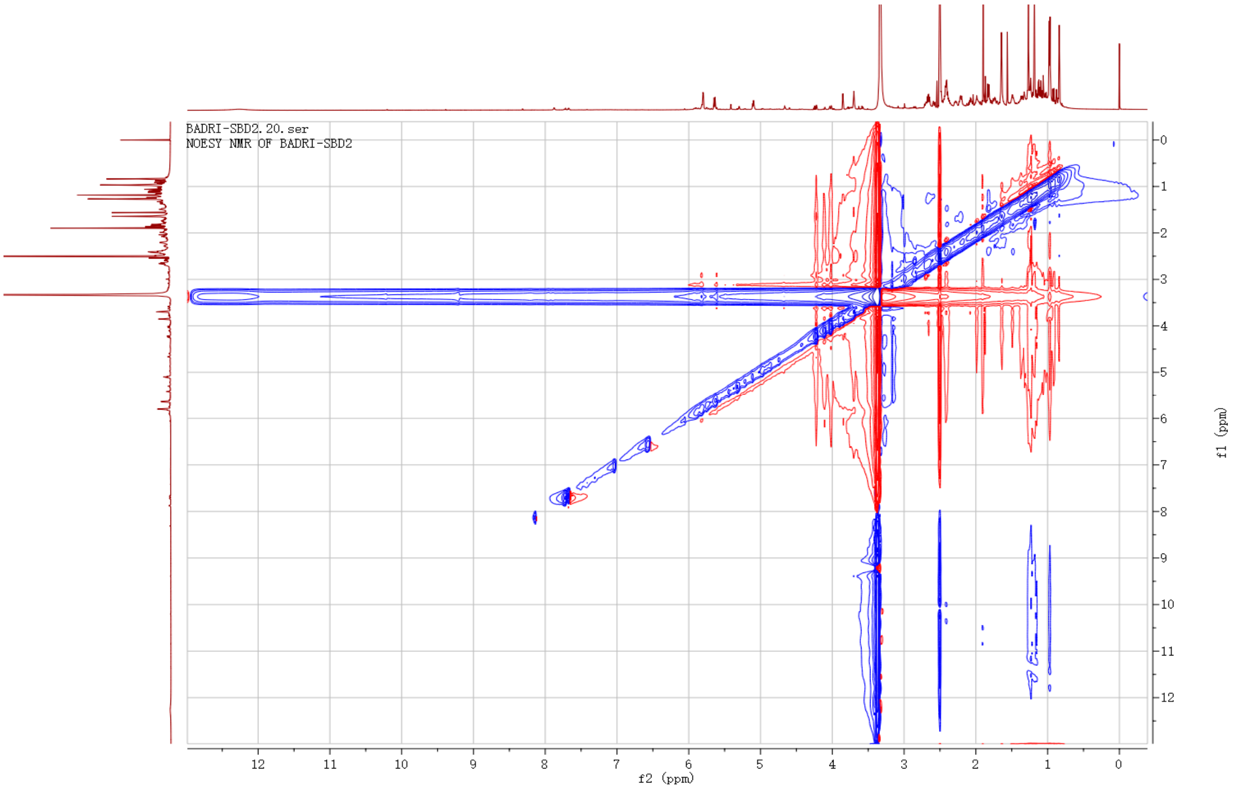

Figure S26. NOESY spectra of the new compound **3.**

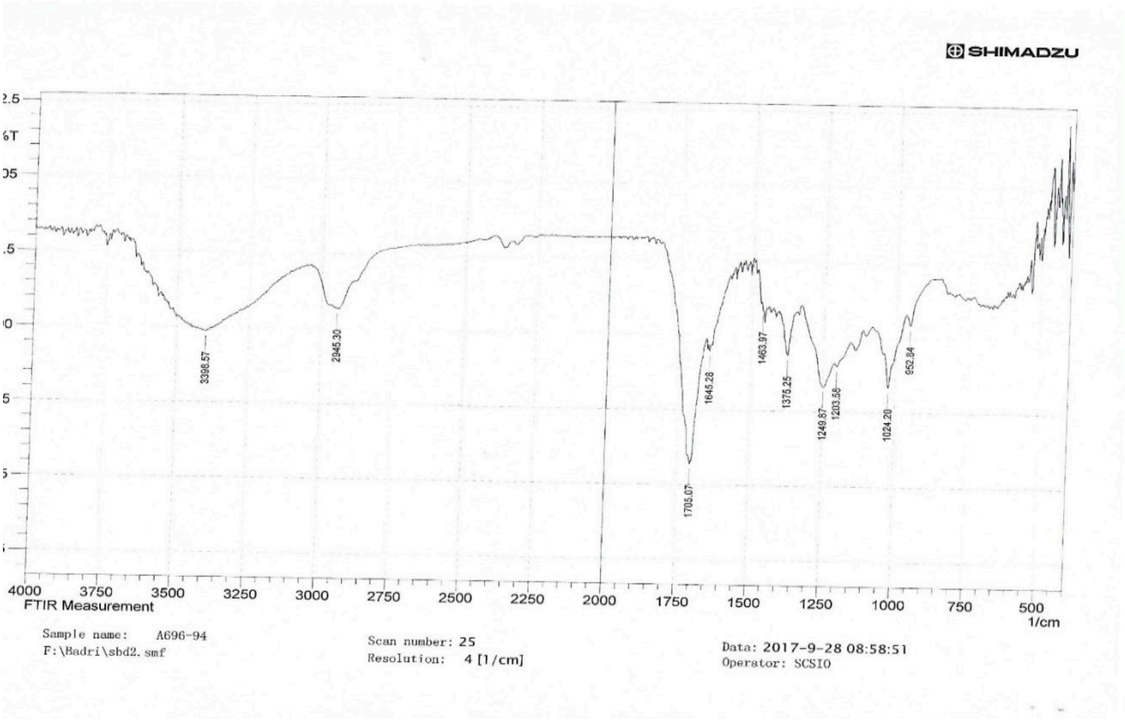

Figure S27. IR spectra of the new compound **3.**

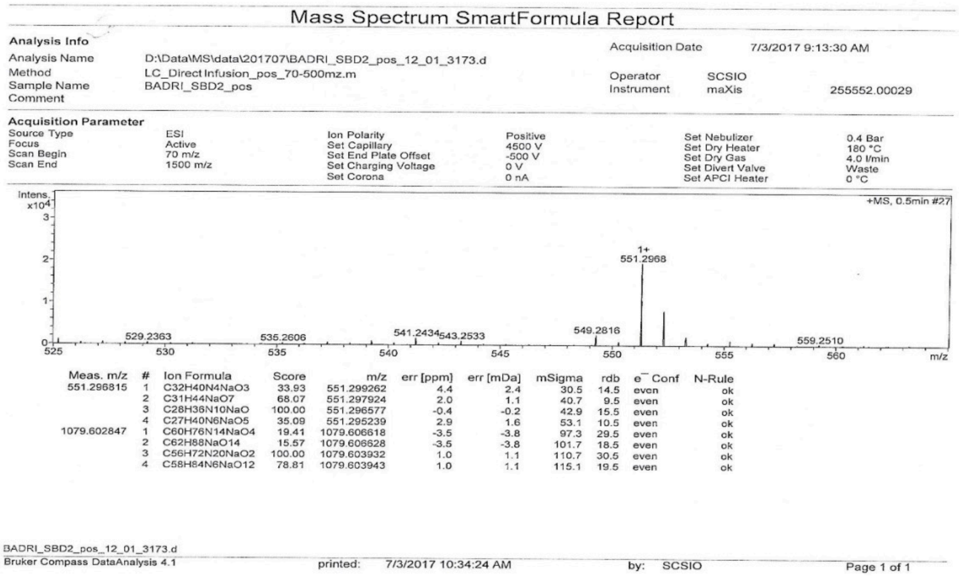

Mass Spectrum SmartFormula Report

Analysis Info

Analysis Name	D:\Data\MS\data\201707\BADRI_SBD2_pos_12_01_3173.d	Acquisition Date	7/3/2017 9:13:30 AM
Method	LC_Direct Infusion_pos_70-500mz.m	Operator	SCSIO
Sample Name	BADRI_SBD2_pos	Instrument	maXis 255552.00029
Comment			

Acquisition Parameter

Source Type	ESI	Ion Polarity	Positive	Set Nebulizer	0.4 Bar
Focus	Active	Set Capillary	4500 V	Set Dry Heater	180 °C
Scan Begin	70 m/z	Set End Plate Offset	-500 V	Set Dry Gas	4.0 l/min
Scan End	1500 m/z	Set Charging Voltage	0 V	Set Divert Valve	Waste
		Set Corona	0 nA	Set APCI Heater	0 °C

Meas. m/z	#	Ion Formula	Score	m/z	err [ppm]	err [mDa]	mSigma	rdb	e⁻ Conf	N-Rule
551.296815	1	C32H40N4NaO3	33.93	551.299262	4.4	2.4	30.5	14.5	even	ok
	2	C31H44NaO7	68.07	551.297924	2.0	1.1	40.7	9.5	even	ok
	3	C28H36N10NaO	100.00	551.296577	-0.4	-0.2	42.9	15.5	even	ok
	4	C27H40N6NaO5	35.09	551.295239	2.9	1.6	53.1	10.5	even	ok
1079.602847	1	C60H76N14NaO4	19.41	1079.606618	-3.5	-3.8	97.3	29.5	even	ok
	2	C62H88NaO14	15.57	1079.606628	-3.5	-3.8	101.7	18.5	even	ok
	3	C56H72N20NaO2	100.00	1079.603932	1.0	1.1	110.7	30.5	even	ok
	4	C58H84N6NaO12	78.81	1079.603943	1.0	1.1	115.1	19.5	even	ok

BADRI_SBD2_pos_12_01_3173.d
Bruker Compass DataAnalysis 4.1 printed: 7/3/2017 10:34:24 AM by: SCSIO Page 1 of 1

Figure S28. HR-ESIMS of the new compound **3.**

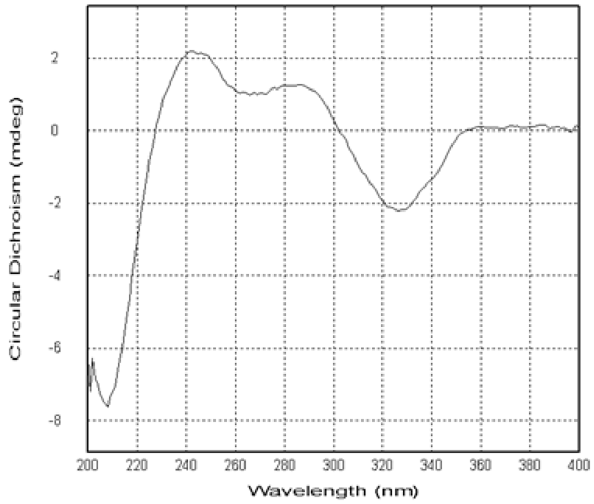

Figure S29. The experimental CD curve of the new compound **3**.

The ITS gene sequence data of *Aspergillus fumigatus* SCSIO 41012

tcttccggatagggggactgCGGAAGGATCATTACCGAGTGAGGGCCCTC
TGGGTCCAACCTCCCACCCGTGTCTATCGTACCTTGTTGCTTCGGCGGGC
CCGCCGTTTCGACGGCCGCCGGGGAGGCCCTGCGCCCCCGGGCCCGCGCC
CGCCGAAGACCCCAACATGAACGCTGTTCTGAAAGTATGCAGTCTGAGTT
GATTATCGTAATCAGTTAAAACTTTCAACAACGGATCTCTTGGTTCCGGC
ATCGATGAAGAACGCAGCGAAATGCGATAAGTAATGTGAATTGCAGAATT
CAGTGAATCATCGAGTCTTTGAACGCACATTGCGCCCCCTGGTATTCCGG
GGGGCATGCCTGTCCGAGCGTCATTGCTGCCCTCAAGCACGGCTTGTGTG
TTGGGCCCCCGTCCCCCTCTCCCGGGGGACGGGCCCGAAAGGCAGCGGCG
GCACCGCGTCCGGTCCTCGAGCGTATGGGGCTTTGTCACCTGCTCTGTAG
GCCCGGCCGGCGCCAGCCGACACCCAACTTTATTTTTCTAAGGTTGACCT
CGGATCAGGTAGGGATACCCGCTGAACTTAAGCATAtcatagccgcgga

ggaa
